# Ethnopharmacological survey of medicinal plants in Jeju Island, Korea

**DOI:** 10.1186/1746-4269-9-48

**Published:** 2013-07-09

**Authors:** Mi-Jang Song, Hyun Kim, Brian Heldenbrand, Jongwook Jeon, Sanghun Lee

**Affiliations:** 1 School of Alternative Medicine and Health Science, Jeonju University, 303 Cheonjam-ro, Jeonju, Wansan-gu, 560-759, Republic of Korea; 2 School of Liberal Arts, Jeonju University, 303 Cheonjam-ro, Jeonju, Wansan-gu, 560-759, Republic of Korea; 3 Korea Institute of Oriental Medicine, 1672 Yuseongdae-ro, Deajeon, Yuseong-gu, 305-811, Republic of Korea

**Keywords:** Traditional Knowledge, Participatory rural appraisal, Informant consensus factor, Fidelity level, Hallasan National Park

## Abstract

**Background:**

This study aims to analyze and record orally transmitted knowledge of medicinal plants from the indigenous people living in Hallasan National Park of Korea.

**Methods:**

Data was collected through the participatory rural appraisal method involving interviews, informal meetings, open and group discussions, and overt observations with semi-structured questionnaires.

**Results:**

In this study, a total of 68 families, 141 genera, and 171 species of plants that showed 777 ways of usage were recorded. Looking into the distribution of the families, 14 species of Asteraceae occupied 11.1% of the total followed by 13 species of Rosaceae, 10 species of Rutaceae, and nine species of Apiaceae which occupied 5.0%, 7.1% and 3.0% of the whole, respectively. 32 kinds of plant-parts were used for 47 various medicinal purposes. Values for the informant consensus factor regarding the ailment categories were for birth related disorders (0.92), followed by respiratory system disorders (0.90), skin disease and disorders (0.89), genitourinary system disorders (0.87), physical pain (0.87), and other conditions. According to fidelity levels, 36 plant species resulted in fidelity levels of 100%.

**Conclusion:**

Consequently, results of this study will legally utilize to provide preparatory measures against the Nagoya Protocol (2010) about benefit-sharing for traditional knowledge of genetic resources.

## Introduction

Hallasan National Park, which possesses a wonderful ecological geography and a unique traditional culture, was designated as a Biosphere Reserve in 2002, a World Natural Heritage in 2007, and a Global Geopark in 2010, making the sub-tropical island the only place on Earth to receive all three United Nations Educational, Scientific and Cultural Organization (UNESCO) designations in the field of natural science.

Mt. Halla (1,950 m) is located at the center of the Hallasan National Park as a volcanic island distributed randomly over 360 parasitic volcanos (“oreums” in Korean). Hallasan National Park is separated by the Jeju Channel, 59 km in width, across from Haenamgot, which is the southernmost tip of the Korean Peninsula and is made up of eight inhabited isles and 54 uninhabited islets. Particularly, Hallasan National Park lies in the middle of the triangle which consists of the Korean Peninsula, the Japanese islands and the Chinese continent. The nearest point to Japan from Jeju Island is the city of Sasebo (250 km); and for China, it is the mouth of the Yangtze River in the Shanghai area. Therefore, this ideal location has been advantageous for exchanging cultures and goods within these regions. Hallasan National Park has been referred to as a small continent in far east Asia due to its unique culture that the people of Jeju have created.

Traditionally, Jeju is famous for its abundance of three items, which include Seokda (rocks), Pungda (wind), and Yeoda (women). Seokda originated from the past volcanic activity of Mt. Halla. The inhabitants of Jeju Island needed to cultivate the land through a long process of clearing away the numerous rocks covering the land and then form inlets for irrigation, and finally construct walls for protection against the wind. The abundance of Seokda speaks of the harsh surroundings on Jeju Island. The island is located in the path of typhoons; therefore, the islanders have had to fight against the sea. The effects of Pungda and Seokda impact the lifestyle of the inhabitants on Jeju Island. Two examples are the thatched roofs which are tied up with straw rope, and the fields surrounded by stone walls. The third element which exists on Jeju Island isYeoda, which originated from the fact that most men on the island were lost at sea, and therefore caused the women to outnumber the men. Also, women had to come out into the fields with men due to the Jeju Island's harsh living environment. The abundance of Yeoda is a stated comment on population statistics, but moreover it is a metaphor for women living on Jeju Island who work diligently. The famous women-divers on the island (“haenyo” in Korean), who fight against the wild waves to catch fish are very symbolic to Jeju Island.

The agriculture of Jeju Island has traditionally been famous for its tangerine orchards and horse breeding due to the fact that the land cannot support rice farming due to the nature of the soil. The weather of Jeju Island depicts a vertical distribution from subtropics to a subarctic zone by its geographical position, its elevation, and topography. Owing to these environmental factors, the vegetation of Hallasan National Park is variously distributed from low-lying warm temperature forests to alpine or arctic forests of its highlands. It has a subtropical evergreen broad-leaved forest zone 600 m above sea level. Also, it has a temperate deciduous broad-leaved forest zone between 600~1,400 m above sea level. And it comprises the vegetation girdle of the subarctic zone or subalpine belt which is between 1,400~1,950 m above sea level. The endemic plants and the diversity of its species are abundant compared to other areas of the Korean Peninsula.

The floral investigation of Hallasan National Park began by Nakai [1], who reported 1,433 species, with both Lee [2] and Park et al. [3] examining the same area. The latest flora count reported 1,800 species by Kim [4] to 1,990 species by Kim et al. [5] in 2006.

The investigation of its medicinal plants began first with 405 species by Do et al. [6]. In 1968, 494 species were found by Do [7], and 425 species were reported by Yuk [8], and in 2004, 801 species were reported by Kim [9]. However, an ethnopharmacological study using orally transmitted traditional knowledge had yet to be considered.

Up to the present, although ethnopharmacological studies on islands of the world has widely been accomplished, such as the Reunion Island [10] of France, three islands on Vanuatu [11], and the Hainan Island of China [12], this research was the first of its kind in Korea and on Jeju Island.

This study aims to record traditional knowledge about medicinal plants orally transmitted from generation to generation in Jeju Island of Korea, where traditional culture and a biogeographic ecosystem, fortunately, have been relatively well conserved.

### Study area and investigative method

### Study area

**Figure 1:**
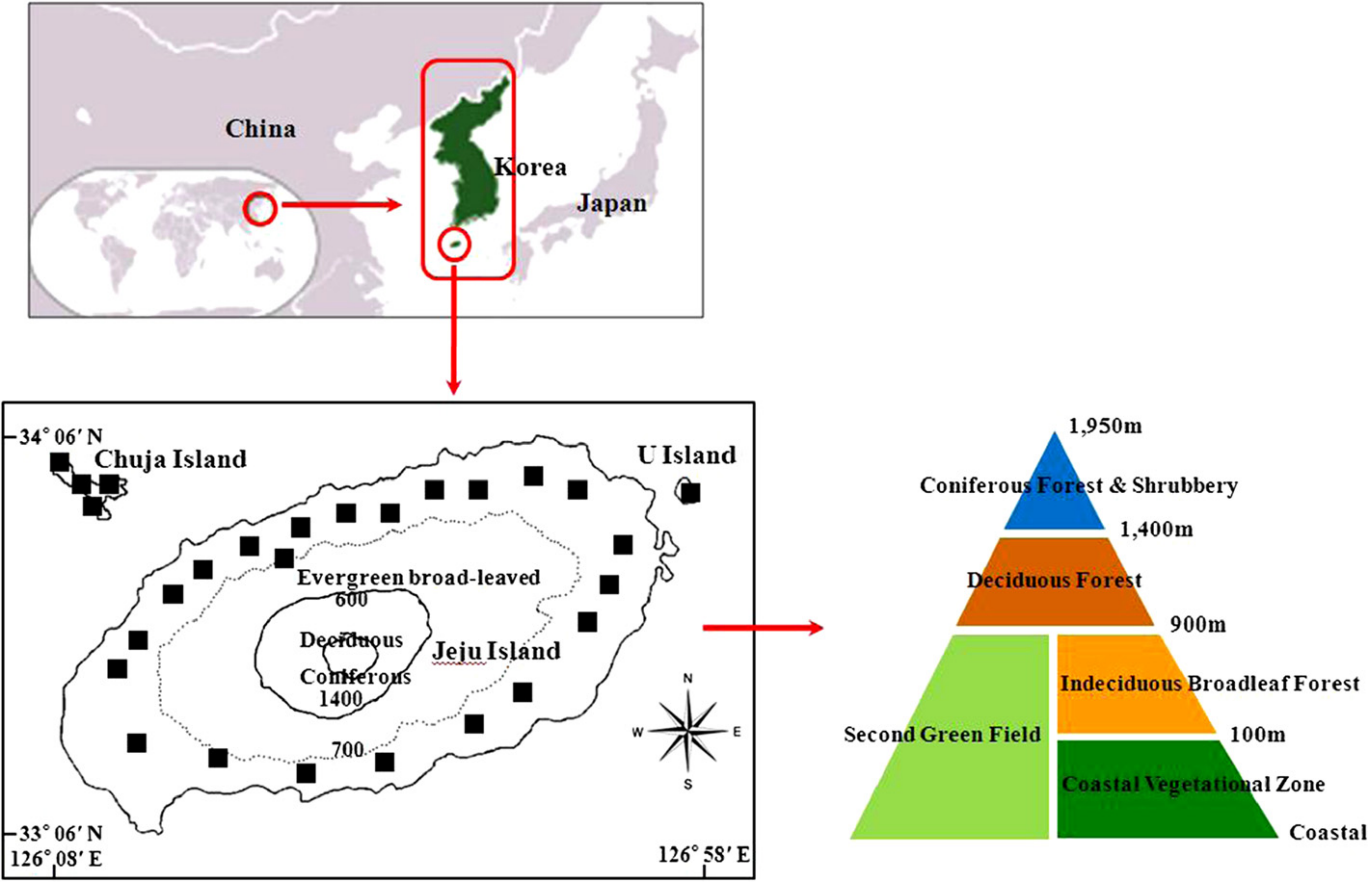
Investigation sites.

The study area is the largest volcanic island in Korea, which lies between 33° 06’N to 34° 00’N latitude and 126° 08’E to 126° 58’E longitude (Figure 1). The entire shape of the island is close to an oval formation in that the major axis inclines at about 15 degrees against the latitude from the northeast to the southwest and it is 2.4 times longer than the minor axis. Its length is 73 km and the width is 41km. The annual average temperature is 15.3°C and the annual precipitation is approximately 1,500~1,600 mm. The study area is divided into two cities, which includes seven counties, five subcounties, and thirty-one villages in its administrative district and measures 1,849.18 km^2^ in area [13]. The total population in 2011 was 583,284 [13].

### Investigative method

Field investigations were conducted throughout 27 sites starting from April, 2011 to November, 2011 (Figure 1). We interviewed 117 key informants who had lived over 40 years in the study area. Proper data was collected using the participatory rural appraisal (PRA) method, as the informants also became investigators themselves, participating in interviews, informal meetings, open and group discussions, and overt observations with semi-structured questionnaires [14-16]. The content of the semi-structured questionnaires was composed of diverse ethnopharmacological information, including local names, plant-parts used, ailments, methods of preparation, manufacturing and administration, dosage, and usable duration regarding each medicine [14-17].

All plant specimens were collected during their flowering or fruiting seasons, and were organized utilizing the normal specimen manufacturing method [14,17]. The voucher specimens were deposited for preservation in the herbarium of Jeonju University. The precise identification of plants mentioned by the informants was performed in accordance with Lee [18] and Lee [19]. Scientific names of plants were confirmed by the National Knowledge and Information System for Biological Species [20] of Korea.

### Quantitative analysis

The informant consensus factor (ICF) was used to identify the ethnopharmacological importance of the collected plant species and to analyze the agreement degree of the informants’ knowledge about each category of ailments [12,21,22]. The ICF was calculated using the following formula: *ICF*=(*n*_*ur*_– *n*_*t*_) / (*n*_*ur*_– 1), where *n*_*ur*_ is the number of times an ailment was mentioned in each category and *n*_*t*_ is the number of plant species used.

The fidelity level (FL) was employed to determine the most important plant species used for treating certain diseases by the local herbal practitioners and elderly people living in the study area [14-16,23]. The FL was calculated using the following formula: *FL(%)=N*_*p*_*× 100 / N*, where *N*_*p*_ is the number of informants that mentioned the specific plant species used to treat certain ailments, and *N* is the total number of the informants who utilized the plants as medicine for treating any given ailment.

## Results and discussion

### Demographic characteristics of participants in the study

All 117 informants (42 men and 75 women) were randomly selected at the community halls, the senior welfare centers, and the traditional markets at 27 designated sites. The average age of the informants was 78 years old with informants ranging in age from 40 to 94. The elderly in their seventies and eighties occupied 82.9% of the total, while 91 informants never received any school education (Table 1).

**Table 1:** Demographic characteristics


Gender	
Male	42 (35.9%)
Female	75 (64.1%)
Age	
40-49	2 (1.7%)
50-59	1 (0.9%)
60-69	10 (8.5%)
70-79	49 (41.9%)
80-89	48 (41.0%)
90-99	7 (6.0%)
Educational attainment	
Never attended school	91 (77.8%)
Attended school for less than 6 years	6 (5.1%)
Attended school for 6 years	7 (6.0%)
Finished middle school	7 (6.0%)
Finished high school	6 (5.1%)

### Medicinal plants and associated knowledge

In this study, a total of 68 families, 141 genera, and 171 species of plants that showed 777 ways of usage were recorded from Hallasan National Park (Additional file 1: Table S1). The recorded plant species totaled 8.6% of the 1,990 species [5] and 21.3% of the 801 medicinal species [9] in the study area. The varying percentage exists for two reasons. One, the local community had not gathered wild plants for usage any longer. Two, most of the elderly people who directly gathered the medicinal plants, had forgotten their preparatory methods and usages. However, the 171 recorded plant species on Jeju Island exceeded the number per square kilometer found on the islands of other countries researched: 75 species found on Reunion Island in France, which is 1.3 times larger in area than Jeju Island [10], 133 species found on the three islands in Vanuatu, which is 6.7 times larger than Jeju Island [11], and 385 species collected on Hainan Island in China, which is 20 times larger than Jeju Island [12].

**Figure 2:**
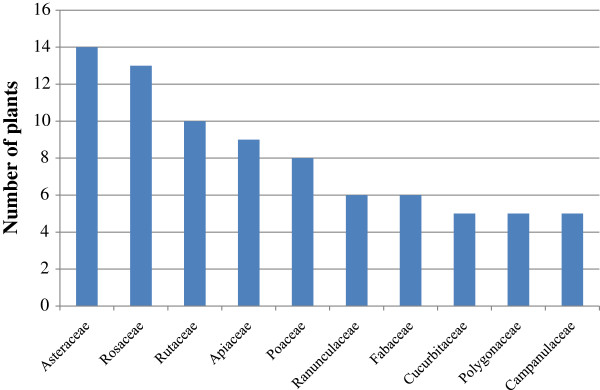
**The most common plant families (56 Outliers omitted found in Additional file****1****: Table S1).**

**Figure 3:**
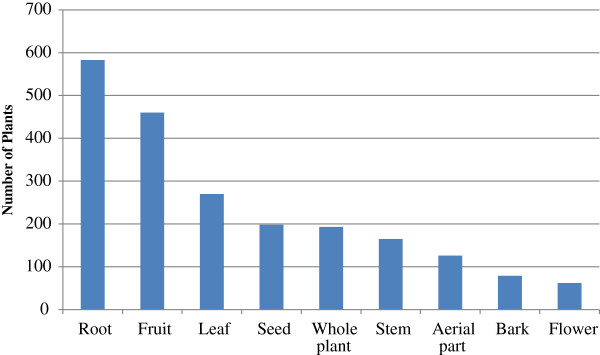
Used plant-parts of medicinal plants.

Looking into the distribution of the families, 14 species of Asteraceae occupied 11.1% of the total followed by 13 species of Rosaceae, 10 species of Rutaceae, and 9 species of Apiaceae, which occupied 5.0%, 7.1% and 3.0% of the whole, respectively (Figure 2). Our analysis reveals that overall, 32 kinds of plant-parts were selected as medicinal materials. Roots were the most frequently used plant-parts, constituting 23.7% of the whole followed by fruits (18.7%), leaves (11%), seeds (8.0%), whole plants (7.8%), stems (6.7%), aerial parts (5.1%), and other sections of the plant (Figure 3). This data was similar to the investigative results of the western plains [16] and the southern mountainous regions [15] of Korea. These results were also similar to other countries including India [24-26], Spain [27] and Brazil [28].

**Figure 4:**
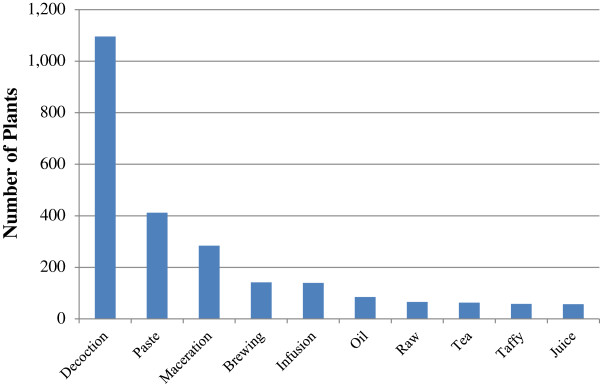
**The most common preparation methods of medicinal plants (37 Outliers omitted found in Additional file****1****: Table S1).**

The results depict 47 modes of preparation for the medicinal materials. Decoctions, pastes, macerations, brewings and infusions occupied 37.5%, 14.1%, 9.7%, 4.9% and 4.8% of the whole, respectively. Oral administration accounted for 73.4% of the applications while topical application results were at 26.4%, while nasal injection completed the list (Figure 4).

**Figure 5:**
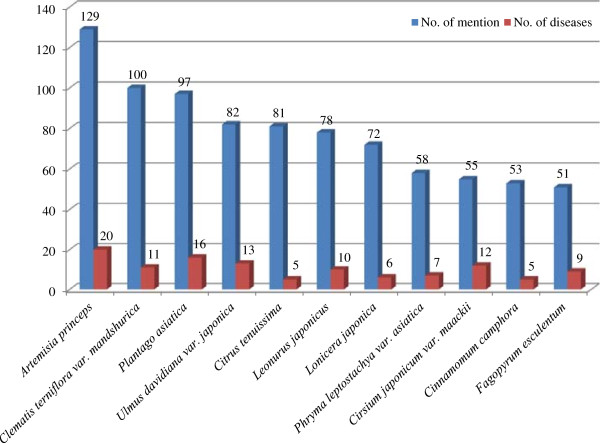
Ailment numbers and medicinal plants mentioned more than 50 times.

Considering the high frequency of medicinal plants mentioned more than 50 times related to medicinal efficacy by the key informants, *Artemisia princeps* Pamp. was used to treat 20 ailments, followed by *Plantago asiatica* L. for treating 16 ailments, *Ulmus davidiana* var. *japonica* (Rehder) Nakai for treating 13 ailments and *Clematis terniflora* var. *mandshurica* (Rupr.) Ohwi for treating 11 ailments (Figure 5). As the key informants continued to use these medicinal plants multiple times for specific ailments with favorable results, these species could be evaluated to possess the function of pharma-foods.

The fruit of *Vitex rotundifolia* L.f. was applied to the pillow for the cure of headaches. The fruit of *Torreya nucifera* (L.) Siebold & Zucc. was used to cure vermicide, as in China and Japan [29-33]. Also, the whole plant of *Phryma leptostachya* var. *oblongifolia* (Koidz.) Honda was utilized as a bath supplement for skin ailments. These medicinal plant species should be developed as health products for their vital contribution in health care and health management.

Particularly, because *Epimedium koreanum* Nakai, which is used as a tonic in far eastern Asia [34], but is not grown in Hallasan National Park, *Caulophyllum robustum* Maxim., *Thalictrum kemense* var. *hypoleucum* (Siebold & Zucc.) Kitag., and *Cimicifuga biternata* Miq. were taken as substitutes. However, the medicinal efficacies of these substitutes are very different from *Epimedium koreanum* Nakai [35]. We believe that the inhabitants of the island used these substitutes and obtained similar psychological benefits.

Finally, we have affirmed that the overall usage pattern of medicinal plants of the inhabitants on Jeju Island is nearly similar to both China and Japan due to the similarity of the flora of medicinal plants.

### Quantitative analysis

#### Informant consensus factor (ICF)

The informant consensus factor (ICF) was used to identify the ethnopharmacological importance of the collected plant species [21,22].

The category with the highest degree of consensus from informants was birth related disorders (0.92). The ranking followed with respiratory system disorders (0.90), skin disease and disorders (0.89), genitourinary system disorders (0.87), physical pain (0.87), and other conditions. The lowest degree of consensus was diabetes (Table 2). These results reflect that in the past the hygienic, climatic and topographical environments of Hallasan National Park were at inferior levels. The high ICF value for respiratory system disorders was due to asthma resulting from an allergic reaction to monstrous mites found in many tangerine orchards [36]. Also, we can conclude that the lowest ICF value for diabetes is due to the coarse food eaten and the harsh living conditions on Jeju Island.

**Table 2:** Category of ailments and their informant consensus factor (ICF) according to Heinrich et al. (1998)

**Symptom and ailment categories**	**TAXONS**	**Use citations**	**ICF**
Birth related disorders	9	100	0.92
Respiratory system disorders	52	533	0.90
Skin disease and disorders	23	209	0.89
Genitourinary system disorders	25	189	0.87
Physical pain	51	376	0.87
Cuts and wounds	15	105	0.87
Inflammation	20	114	0.83
Gastrointestinal disorders	56	322	0.83
Others	45	226	0.80
Poisonings	11	52	0.80
Nervous system disorders	34	141	0.76
Muscular-skeletal disorders	16	48	0.68
Circulatory system disorders	32	97	0.68
Liver complaints	14	39	0.66
Diabetes	12	25	0.54

### Fidelity level (FL)

The fidelity level is useful for identifying the inhabitants’ most preferred species in use for treating certain ailments [23]. FL values in this study varied from 1.0% to 100%. Generally, a FL of 100% for a specific plant indicates that all of the use-reports mentioned the same method for using the plant for treatment [37]. The study determined 36 species of plants with a FL of 100%, even without considering plants that were mentioned only once for better accuracy (Additional file 1: Table S1). This information means that the informants had a tendency to rely on one specific plant species for treating one certain ailment than for several ailments.

With special attention given to important species (N, Np) of plants with an FL above 90% regarding the viewpoint of the number of times mentioned and the consensus level for the specific ailment, *Citrus tenuissima* Tanaka. (81, 61), *Pyrus pyrifolia* Nakai (41, 34), *Cimicifuga heracleifolia* Kom.(14, 12) and *Citrus aurantium* L. (11, 11) were used to treat the common cold, respectively. Also, *Cinnamomum camphora* (L.) J. Presl (53, 48) was used for various cancers, *Undaria pinnatifida* (Harvey) Suringar (28, 24) for Puerperalism, *Torreya nucifera* (L.) Siebold & Zucc. (18, 18) for parasites, *Solanum tuberosum* L. (19, 17) for burns, *Imperata cylindrica* var. *koenigii* (Benth.) Druce (18, 16) for snakebites, *Papaver somniferum* L. (15, 12) for furuncle, and *Potentilla chinensis* Ser. var. *chinensis* (11, 11) for tingling (Additional file 1: Table S1).

### Review of local plant names

The local plant names occasionally had information for understanding the properties of the medicinal plants. The pronunciation and meaning of dialectics, including the plant names of Hallasan National Park, were considerably different from standard Korean. The local plant names of Hallasan National Park were investigated by Nakai [1] and Kim [38]. The unique characteristics of the local plant names were also confirmed in this study. Namely, the phonemes of classic Korean in the 15th century have uniquely remained in the names of 25 plant species even to this day (Table 3).

**Table 3:** Phonemes of classic Korean in the 15th century that uniquely remain in the names of 25 species

**Scientific name**	**Standard Korean name**	**Local name on Jeju Island**
*Achyranthes japonica* (Miq.) Nakai	Soemureup	
*Arisaema amurense* for. *serratum* (Nakai) Kitag.	Cheonnamseong	
*Breea segeta* (Willd.) Kitam. f. *segeta*	Jobaengi	
*Cirsium japonicum* var. *maackii* (Maxim.) Matsum.	Eonggeongkwi	
*Citrus juno*s Siebold ex Tanaka	Yujanamu	
*Citrus tenuissima* Tanaka.	Dangyujanamu	
*Euonymus alatus* (Thunb.) Siebold	Hwasalnamu	
*Euscaphis japonic*a (Thunb.) Kanitz	Malojumttae	
*Fagopyrum esculentum* Moench	Memil	
*Gardenia jasminoides* Ellis	Chijanamu	
*Lagenaria leucantha* Rusby	Bak	
*Luffa cylindrica* Roem.	Susemioi	
*Melia azedarach* L.	Meolguseulnamu	
*Polygonum aviculare* L.	Madipul	
*Poncirus trifoliata* Raf.	Taengjanamu	
*Prunus tomentosa* Thunb.	Aengdonamu	
*Raphanus sativus* L.	Mu	
*Ricinus communis* L.	Pimaja	
*Schisandra chinensis* (Turcz.) Baill.	Omija	
*Solanum nigrum* L. var. *nigrum*	Kkamajung	
*Sophora flavescens* Solander ex Aiton	Gosam	
*Torreya nucifera* (L.) Siebold & Zucc.	Bijanamu	
*Viola mandshurica* W. Becker	Jebikkot	
*Zanthoxylum piperitum* (L.) DC.	Chopinamu	
*Zanthoxylum planispinum* Siebold & Zucc.	Gaesancho	

## Conclusion

Hallasan National Park has been designated as a cultural, topological, and natural heritage of the world by UNESCO, as it lies in the middle of the triangle which makes up the Korean Peninsula, the Japanese islands and the Chinese continent, and for being home to various plants which contain interesting properties according to an ethnopharmacological viewpoint.

Particularly, the characteristics of traditional ailments and the use of medicinal plants of Hallasan National Park have been brought to light. First, the traditional ailments of the local communities were evaluated by both climatic and geoecological environments. The respiratory ailments of Hallasan National Park were much higher than any other region because of windy and humid conditions. Second, ailments due to traditional occupations also existed, like cases of arthritis for the women divers in Jeju Island. Third, people used medicinal plants of similar shape for the same purpose, even though they had a different efficacy (for example, *Epimedium koreanum* Nakai). These properties need further study using an investigative method in social medicine for a more exact analysis.

Also, medicinal plants, including *Lagenaria leucantha* Rusby, *Citrus aurantium* L., *Trichosanthes kirilowii* var. *japonica* Kitam., *Neolitsea sericea* (Blume) Koidz., *Duchesnea indica* (Andrews) Focke, and *Phryma leptostachya* var. *oblongifolia* (Koidz.) Honda were mentioned significantly and have high FL values in categories of a high ICF index. These species will be able to develop as pharmafoods or pharmaceuticals.

However, it is expected that the rapid decrease of the senior population which directly gathers wild medicinal plants will certainly lead to a greater loss of oral traditional knowledge similar to other regions in Korea [15,16].

We keenly realize the necessity for a sustainable conservation of orally transmitted traditional knowledge of medicinal plants.

## Competing interests

The authors declare that they have no competing interests.

## Authors’ contribution

HK and MJS complied the collected field data, analyzed and drafted the manuscript, BH, JWJ, and SHL revised the manuscript and added the valuable suggestions for improving the manuscript. All authors read and approved the final manuscript.

## Supplementary Material

Additional file 1: Table S1. Plant species used to treat ailments (Scientific names according to the international names index). Click here for file
