# Supplementary material for: Ethnopharmacological survey of medicinal plants in Jeju Island, Korea
Source: J Ethnobiol Ethnomed. 2013 Jul 9;9:48. doi: 10.1186/1746-4269-9-48 (PMC3716930; doi:10.1186/1746-4269-9-48)
Supplement: Additional file 1: Table S1. — Plant species used to treat ailments (Scientific names according to the international names index). [file 1746-4269-9-48-S1.docx]

| Scientific name | Voucher | Family | Korean name | Used part | Ailments | Preparation | Application | FL |
| --- | --- | --- | --- | --- | --- | --- | --- | --- |
| *Achyranthes japonica* (Miq.) | KH5023 | Amaranthaceae | Soemureup | Root | Bone disease | Decoction | Oral | 10.5 |
| Nakai |  |  |  |  |  | Taffy | Oral |  |
|  |  |  |  |  | Knee pain | Brewing | Oral | 55.3 |
|  |  |  |  |  |  | Decoction | Oral |  |
|  |  |  |  |  |  | Maceration, paste | Topical |  |
|  |  |  |  |  | Neuralgia | Decoction | Oral | 34.2 |
|  |  |  |  |  |  | Taffy | Oral |  |
|  |  |  |  | Stem | Neuralgia | Decoction | Oral |  |
| *Aconitum ciliare* DC. | KH5024 | Ranunculaceae | Notjeotgaraknamul | Root | Lumbago | Decoction | Oral | 50.0 |
|  |  |  |  |  | Neuralgia | Decoction | Oral | 50.0 |
| *Aconitum coreanum* H. Lév | KH5025 | Ranunculaceae | Baekbuja | Root | Fatigue | Decoction | Oral | 100.0 |
| *Acorus gramineus* Aiton | KH5026 | Acoraceae | Seokchangpo | Root | Neurasthenia | Decoction | Oral | 100.0 |
| *Adenophora thunbergiana* | KH5027 | Campanulaceae | Jandae | Root | Blood circulation | Decoction | Oral | 33.3 |
| Kudo |  |  |  |  | Enterotoxin | Warm up in a double | Oral | 33.3 |
|  |  |  |  |  |  | boiler |  |  |
|  |  |  |  |  | Leg pain | Decoction | Oral | 33.3 |
| *Allium cepa* L. | KH5028 | Alliaceae | Yangpa | Bulb | Common cold | Tea | Oral | 100.0 |
| *Allium fistulosum* L. | KH5029 | Alliaceae | Pa | Whole plant | Cold and flu | Decoction | Oral | 22.2 |
|  |  |  |  |  | Common cold | Decoction | Oral | 77.8 |
|  |  |  |  |  |  | Raw | Oral |  |
| *Allium scorodoprasum* | KH5030 | Alliaceae | Maneul | Bulb | Abdominal pain | Juice | Oral | 40.0 |
| var. *multibulbillosum* |  |  |  |  | Cold and flu | Decoction | Oral | 13.3 |
| Y.N.Lee |  |  |  |  | Cradle cap | Maceration, rubbing | Topical | 13.3 |
|  |  |  |  |  | Furuncle | Juice, applying | Topical | 13.3 |
|  |  |  |  |  | Gastroenteric | Decoction | Oral | 20.0 |
|  |  |  |  |  | disorder |  |  |  |
| *Allium tuberosum* Rottler ex | KH5031 | Alliaceae | Buchu | Aerial part | Dental caries | Roll in salt, hold it | Topical | 9.1 |
| Spreng. |  |  |  |  |  | between one's |  |  |
|  |  |  |  |  |  | teeth |  |  |
|  |  |  |  |  | Dental pain | Maceration, hold it | Topical | 54.5 |
|  |  |  |  |  |  | between one's teeth |  |  |
|  |  |  |  |  |  | Preserve in salt, hold it | Topical |  |
|  |  |  |  |  |  | between one's teeth |  |  |
|  |  |  |  |  | Periodontitis | Preserve in salt, paste | Topical | 18.2 |
|  |  |  |  |  | Stomatitis | Coated with salt, | Topical | 18.2 |
|  |  |  |  |  |  | stirring |  |  |
|  |  |  |  |  | Stomatitis | Preserve in salt, paste | Topical |  |
| *Allium wakegi* Araki | KH5032 | Alliaceae | Jjokpa | Whole plant | Common cold | Decoction | Oral | 100.0 |
| *Althaea rosea* Cav. | KH5033 | Malvaceae | Jeopsikkot | Root | Spontaneous | Decoction | Oral | 25.0 |
|  |  |  |  |  | abortion |  |  |  |
|  |  |  |  |  | Sterility | Decoction | Oral | 75.0 |
| *Angelica dahurica* (Hoffm.) | KH5034 | Apiaceae | Guritdae | Root | Edema | Decoction | Oral | 33.3 |
| Benth. & Hook. f. ex |  |  |  |  | Neuralgia | Decoction | Oral | 66.7 |
| Franch. & Sav. |  |  |  |  |  |  |  |  |
| *Angelica gigas* Nakai | KH5035 | Apiaceae | Chamdanggwi | Root | Blood circulation | Decoction | Oral | 33.3 |
|  |  |  |  |  | Common cold | Decoction | Oral | 16.7 |
|  |  |  |  |  | Spontaneous | Decoction | Oral | 16.7 |
|  |  |  |  |  | abortion |  |  |  |
|  |  |  |  |  | Sterility | Decoction | Oral | 16.7 |
|  |  |  |  |  | Woman disease | Decoction | Oral | 16.7 |
| *Aralia elata* (Miq.) Seem. | KH5036 | Araliaceae | Dureupnamu | Root | Glycosuria | Decoction | Oral | 100.0 |
| *Arisaema amurense* for*.* | KH5037 | Araceae | Cheonnamseong | Root | Afterpain | Decoction | Oral | 11.1 |
| *serratum* (Nakai) Kitag. |  |  |  |  | Bone disease | Brewing | Oral | 7.4 |
|  |  |  |  |  | Bruise | Brewing | Oral | 37.0 |
|  |  |  |  |  |  | Clear soup with | Oral |  |
|  |  |  |  |  |  | dumplings |  |  |
|  |  |  |  |  |  | Decoction | Oral |  |
|  |  |  |  |  | Chronic myofascial | Brewing | Oral | 40.7 |
|  |  |  |  |  | pain |  |  |  |
|  |  |  |  |  |  | Clear soup with | Oral |  |
|  |  |  |  |  |  | dumplings |  |  |
|  |  |  |  |  |  | Decoction | Oral |  |
|  |  |  |  |  | Lumbago | Clear soup with | Oral | 3.7 |
|  |  |  |  |  |  | dumplings |  |  |
| *Artemisia princeps* Pamp. | KH5038 | Asteraceae | Ssuk | Aerial part | Abdominal pain | Juice | Oral | 6.2 |
|  |  |  |  |  | Hemostasis | Maceration, paste | Topical | 20.9 |
|  |  |  |  |  | Knee pain | Moxibustion | Topical | 4.7 |
|  |  |  |  |  | Postpartum care | Infusion, bath | Topical | 18.6 |
|  |  |  |  | Leaf | Abdominal pain | Juice | Oral |  |
|  |  |  |  |  |  | Tea | Oral |  |
|  |  |  |  |  | Bee sting | Rubbing, paste | Topical | 1.6 |
|  |  |  |  |  | Bloody discharge | Decoction | Oral | 1.6 |
|  |  |  |  |  | Bruise | Rubbing, paste | Topical | 3.9 |
|  |  |  |  |  | Cancer | Decoction | Oral | 1.6 |
|  |  |  |  |  | Diarrhea | Extraction | Oral | 7.0 |
|  |  |  |  |  |  | Juice | Oral |  |
|  |  |  |  |  |  | Moxibustion | Topical |  |
|  |  |  |  |  | Dysentery | Moxibustion | Topical | 2.3 |
|  |  |  |  |  | Gastroenteric | Decoction | Oral | 1.6 |
|  |  |  |  |  | disorder |  |  |  |
|  |  |  |  |  | Hemostasis | Maceration, paste | Topical | 20.9 |
|  |  |  |  |  |  | Rubbing, paste | Topical |  |
|  |  |  |  |  | Incised wound | Rubbing, paste | Topical | 0.8 |
|  |  |  |  |  | Irregular | Decoction | Oral | 1.6 |
|  |  |  |  |  | menstruation |  |  |  |
|  |  |  |  |  | Knee pain | Moxibustion | Topical | 4.7 |
|  |  |  |  |  |  | Poultice | Topical |  |
|  |  |  |  |  | Lumbago | Moxibustion | Topical | 1.6 |
|  |  |  |  |  | Physical pain | Moxibustion | Topical | 1.6 |
|  |  |  |  |  | Panacea | Decoction | Oral | 1.6 |
|  |  |  |  |  | Postpartum care | Infusion, bath | Topical | 18.6 |
|  |  |  |  |  | Skin disease | Decoction, wash | Topical | 17.1 |
|  |  |  |  |  |  | Infusion, bath | Topical |  |
|  |  |  |  |  | Woman disease | Poultice | Topical | 1.6 |
|  |  |  |  | Whole plant | Postpartum care | Infusion, bath | Topical |  |
|  |  |  |  |  | Skin disease | Infusion, bath | Topical |  |
|  |  |  |  | Young leaf | Abdominal pain | Infusion | Oral |  |
|  |  |  |  |  | Furuncle | Infusion, bath | Topical | 0.8 |
|  |  |  |  |  | Gastralgia | Juice | Oral | 3.9 |
|  |  |  |  |  | Postpartum care | Infusion, bath | Topical |  |
|  |  |  |  |  | Skin disease | Infusion, Bath | Topical |  |
| *Asarum sieboldii* Miq. | KH5039 | Aristolochiaceae | Jokdoripul | Root | Abortion | Decoction | Oral | 25.0 |
|  |  |  |  |  | Blood circulation | Decoction | Oral | 25.0 |
|  |  |  |  |  | Halitosis | Decoction, mouth | Topical | 25.0 |
|  |  |  |  |  |  | wash |  |  |
|  |  |  |  |  | Sterility | Decoction | Oral | 25.0 |
| *Asparagus cochinchinensis* | KH5040 | Asparagaceae | Cheonmundong | Root | Asthma | Decoction | Oral | 50.0 |
| Merr. |  |  |  |  | Cough | Decoction | Oral | 50.0 |
| *Aster scaber* Thunb. | KH5041 | Asteraceae | Chamchwi | Leaf | Jaundice | Parboil | Oral | 100.0 |
| *Atractylodes ovata* DC. | KH5042 | Asteraceae | Sapju | Root | Lumbago | Decoction | Oral | 50.0 |
|  |  |  |  |  | Neuralgia | Decoction | Oral | 50.0 |
| *Boehmeria nivea* Gaudich. | KH5043 | Urticaceae | Mosipul | Root | Induced abortion | Juice | Oral | 83.3 |
|  |  |  |  |  | Knee pain | Maceration, paste | Topical | 16.7 |
| *Breea segetum* (Bunge) | KH5044 | Asteraceae | Jobaengi | Root | Liver cancer | Decoction | Oral | 100.0 |
| Kitam. |  |  |  |  |  |  |  |  |
| *Bupleurum falcatum* L. | KH5045 | Apiaceae | Siho | Root | Common cold | Decoction | Oral | 40.0 |
|  |  |  |  |  | Liver cirrhosis | Decoction | Oral | 20.0 |
|  |  |  |  |  | Tonic | Decoction | Oral | 40.0 |
| *Bupleurum bicaule* Helm | KH5046 | Apiaceae | Chamsiho | Whole plant | Liver cancer | Decoction | Oral | 100.0 |
| *Camellia japonica* L. | KH5047 | Theaceae | Dongbaeknamu | Fruit | Abdominal pain | Oil | Oral | 6.9 |
|  |  |  |  |  | Asthma | Oil | Oral | 17.2 |
|  |  |  |  |  | Bronchitis | Oil | Oral | 17.2 |
|  |  |  |  |  | Common cold | Oil | Oral | 3.4 |
|  |  |  |  |  | Constipation | Oil | Oral | 6.9 |
|  |  |  |  |  | Cough | Oil | Oral | 31.0 |
|  |  |  |  |  | Lung disease | Oil | Oral | 17.2 |
| *Celtis sinensis* Pers. | KH5048 | Ulmaceae | Paengnamu | Fruit | Fishbone stuck in | Powder | Oral | 75.0 |
|  |  |  |  |  | throat | Raw | Oral |  |
|  |  |  |  |  | Indigestion | Powder | Oral | 25.0 |
|  |  |  |  | Stem | Fishbone stuck in | Raw | Raw |  |
|  |  |  |  |  | throat |  |  |  |
| *Centella asiatica* (L.) Urb. | KH5049 | Apiaceae | Byeongpul | Leaf | Boil | Beating, paste | Topical | 66.7 |
|  |  |  |  |  | Furuncle | Paste | Topical | 33.3 |
| *Cimicifuga biternata* Miq. | KH5050 | Ranunculaceae | Gaeseungma | Whole plant | Common cold | Decoction | Oral | 25.0 |
|  |  |  |  |  | Sexual enhancement | Boiling | Oral | 25.0 |
|  |  |  |  |  | Spontaneous | Decoction | Oral | 25.0 |
|  |  |  |  |  | abortion |  |  |  |
|  |  |  |  |  | Sterility | Decoction | Oral | 25.0 |
| *Cimicifuga heracleifolia* | KH5051 | Ranunculaceae | Seungma | Root | Afterpain | Decoction | Oral | 14.3 |
| Kom. |  |  |  |  | Common cold | Decoction | Oral | 85.7 |
| *Cinnamomum camphora* (L.) | KH5052 | Lauraceae | Noknamu | Bark | Cancer | Decoction | Oral | 90.6 |
| J. Presl |  |  |  |  |  | Tea | Oral |  |
|  |  |  |  |  | Panacea | Decoction | Oral | 3.8 |
|  |  |  |  | Inner layer | Cancer | Decoction | Oral |  |
|  |  |  |  | of bark |  |  |  |  |
|  |  |  |  | Leaf | Cancer | Decoction | Oral |  |
|  |  |  |  |  |  | Tea | Oral |  |
|  |  |  |  | Root | Cancer | Decoction | Oral |  |
|  |  |  |  |  |  | Tea | Oral |  |
|  |  |  |  | Root bark | Cancer | Decoction | Oral |  |
|  |  |  |  |  | Inflammation | Maceration, paste | Topical | 1.9 |
|  |  |  |  |  | Pus | Maceration, paste | Topical | 1.9 |
|  |  |  |  |  | Pyopoiesis | Maceration, paste | Topical | 1.9 |
|  |  |  |  | Stem | Cancer | Decoction | Oral |  |
|  |  |  |  |  |  | Tea | Oral |  |
| *Cinnamomum loureiroi* Nees | KH5053 | Lauraceae | Yukgyenamu | Bark | Blood circulation | Tea | Oral | 100.0 |
| *Cirsium japonicum* var. | KH5054 | Asteraceae | Eonggeongkwi | Root | Bone disease | Taffy | Oral | 3.6 |
| *maackii* (Maxim.) Matsum. |  |  |  |  | Cancer | Decoction | Oral | 3.6 |
|  |  |  |  |  | Cough | Brewing | Oral | 7.3 |
|  |  |  |  |  |  | Taffy | Oral |  |
|  |  |  |  |  | Gastroenteric | Decoction | Oral | 3.6 |
|  |  |  |  |  | disorder |  |  |  |
|  |  |  |  |  | Induced abortion | Juice | Oral | 5.5 |
|  |  |  |  |  | Knee pain | Decoction | Oral | 16.4 |
|  |  |  |  |  |  | Decoction, poultice | Topical |  |
|  |  |  |  |  |  | Maceration, paste | Topical |  |
|  |  |  |  |  | Leg pain | Infusion | Oral | 5.5 |
|  |  |  |  |  |  | Simmer | Oral |  |
|  |  |  |  |  | Liver cancer | Decoction | Oral | 1.8 |
|  |  |  |  |  | Lumbago | Infusion | Oral | 5.5 |
|  |  |  |  |  |  | Maceration, paste | Topical |  |
|  |  |  |  |  | Neuralgia | Brewing | Oral | 40.0 |
|  |  |  |  |  |  | Decoction | Oral |  |
|  |  |  |  |  |  | Taffy | Oral |  |
|  |  |  |  |  | Nosebleed | Decoction | Oral | 3.6 |
|  |  |  |  |  | Panacea | Decoction | Oral | 3.6 |
| *Cirsium japonicum* | KH5055 | Asteraceae | Gasieonggeongkwi | Root | Carpal tunnel | Decoction | Oral | 30.8 |
| var. *spinossimum* (Kitam.) |  |  |  |  | syndrome |  |  |  |
| Kitam. |  |  |  |  | Intestinal disease | Decoction | Oral | 7.7 |
|  |  |  |  |  | Lumbago | Brewing | Oral | 30.8 |
|  |  |  |  |  |  | Decoction | Oral |  |
|  |  |  |  |  | Nosebleed | Brewing | Oral | 30.8 |
|  |  |  |  |  |  | Decoction | Oral |  |
| *Cirsium pendulum* Fisch. ex | KH5056 | Asteraceae | Keuneonggeongkwi | Root | Lumbago | Brewing | Oral | 50.0 |
| DC. |  |  |  |  |  | Decoction | Oral |  |
|  |  |  |  |  | Nosebleed | Brewing | Oral | 50.0 |
|  |  |  |  |  |  | Decoction | Oral |  |
| *Citrus aurantium* L. | KH5057 | Rutaceae | Gwanggyul | Pericarp | Common cold | Decoction | Oral | 84.6 |
|  |  |  |  |  | Cough | Decoction | Oral | 7.7 |
|  |  |  |  |  | Sputum | Decoction | Oral | 7.7 |
| *Citrus hassaku* Hort. ex Yu. | KH5058 | Rutaceae | Palsak | Pericarp | Common cold | Tea | Oral | 100.0 |
| Tanaka |  |  |  |  |  |  |  |  |
| *Citrus junos* Siebold ex | KH5059 | Rutaceae | Yujanamu | Fruit | Common cold | Decoction | Oral | 75.0 |
| Tanaka |  |  |  |  |  | Extraction | Oral |  |
|  |  |  |  |  |  | Simmer | Oral |  |
|  |  |  |  |  |  | Tea | Oral |  |
|  |  |  |  |  | Cough | Decoction | Oral | 10.0 |
|  |  |  |  |  | Liver disease | Decoction | Oral | 15.0 |
|  |  |  |  | Pericarp | Common cold | Tea | Oral |  |
| *Citrus natsudaidai* Hayata | KH5060 | Rutaceae | Hagyul | Fruit | Common cold | Decoction | Oral | 66.7 |
|  |  |  |  |  |  | Extraction, tea | Oral |  |
|  |  |  |  |  | Liver disease | Juice | Oral | 33.3 |
|  |  |  |  | Pericarp | Common cold | Decoction | Oral |  |
| *Citrus tenuissima* Tanaka. | KH5061 | Rutaceae | Dangyujanamu | Fruit | Bronchitis | Decoction | Oral | 4.9 |
|  |  |  |  |  | Cold and flu | Decoction | Oral | 2.5 |
|  |  |  |  |  | Common cold | Decoction | Oral | 75.3 |
|  |  |  |  |  |  | Extraction, tea | Oral |  |
|  |  |  |  |  | Cough | Decoction | Oral | 11.1 |
|  |  |  |  |  | Liver disease | Decoction | Oral | 6.2 |
|  |  |  |  | Pericarp | Common cold | Decoction | Oral |  |
| *Citrus unshiu* (Swingle) | KH5062 | Rutaceae | Gyul | Pericarp | Common cold | Decoction | Oral | 100.0 |
| Marcov. |  |  |  |  |  | Tea | Oral |  |
| *Clematis terniflora* | KH5063 | Ranunculaceae | Euari | Leaf | Facial nerve | Maceration, paste | Topical | 1.0 |
| var. *mandshurica* (Rupr.) |  |  |  |  | paralysis |  |  |  |
| Ohwi |  |  |  |  | Knee pain | Poultice | Topical | 11.0 |
|  |  |  |  |  | Lumbago | Juice | Oral | 54.0 |
|  |  |  |  |  |  | Maceration, paste | Topical |  |
|  |  |  |  |  |  | Poultice | Topical |  |
|  |  |  |  |  |  | Raw, paste | Topical |  |
|  |  |  |  |  | Neuralgia | Maceration, paste | Topical | 10.0 |
|  |  |  |  | Root | Gastritis | Taffy | Oral | 4.0 |
|  |  |  |  |  | Gastroenteric | Decoction | Oral | 3.0 |
|  |  |  |  |  | disorder |  |  |  |
|  |  |  |  |  | Gout | Pill | Oral | 1.0 |
|  |  |  |  |  | Knee pain | Maceration, paste | Topical |  |
|  |  |  |  |  |  | Taffy | Oral |  |
|  |  |  |  |  | Leg pain | Maceration, paste | Topical | 4.0 |
|  |  |  |  |  | Lumbago | Decoction | Oral |  |
|  |  |  |  |  |  | Maceration, paste | Topical |  |
|  |  |  |  |  | Neuralgia | Decoction | Oral |  |
|  |  |  |  |  |  | Maceration, paste | Topical |  |
|  |  |  |  |  |  | Taffy | Oral |  |
|  |  |  |  |  | Tonsillitis | Decoction | Oral | 1.0 |
|  |  |  |  | Stem | Lumbago | Juice | Oral |  |
|  |  |  |  |  |  | Maceration, paste | Topical |  |
|  |  |  |  |  |  | Raw, paste | Topical |  |
|  |  |  |  |  | Neuralgia | Maceration, paste | Topical |  |
|  |  |  |  |  | Skin disease | Infusion, wash | Topical | 3.0 |
|  |  |  |  | Whole plant | Bone disease | Maceration, paste | Topical | 8.0 |
|  |  |  |  |  | Lumbago | Decoction | Oral |  |
|  |  |  |  |  |  | Maceration, paste | Topical |  |
| *Clerodendrum trichotomum* | KH5064 | Lamiaceae | Nurijangnamu | Leaf | Bone disease | Beating, paste | Topical | 19.0 |
| Thunb. |  |  |  |  | Knee pain | Maceration, paste | Topical | 14.3 |
|  |  |  |  |  | Pus | Beating, paste | Topical | 23.8 |
|  |  |  |  | Root | Arthritis | Maceration, paste | Topical | 23.8 |
|  |  |  |  |  | Gout | Decoction | Oral | 19.0 |
| *Cnidium officinale* Makino | KH5065 | Apiaceae | Cheongung | Root | Spontaneous | Decoction | Oral | 50.0 |
|  |  |  |  |  | abortion |  |  |  |
|  |  |  |  |  | Sterility | Decoction | Oral | 50.0 |
| *Codonopsis lanceolata* | KH5066 | Campanulaceae | Deodeok | Root | Asthma | Decoction | Oral | 20.0 |
| Trautv. |  |  |  |  | Common cold | Decoction | Oral | 60.0 |
|  |  |  |  |  | Sthenia | Infusion | Oral | 20.0 |
| *Codonopsis ussuriensis* | KH5067 | Campanulaceae | Sogyeongbulal | Root | Asthma | Brewing | Oral | 50.0 |
| (Rupr. & Maxim.) Hemsl. |  |  |  |  |  | Fermentation | Oral |  |
|  |  |  |  |  |  | Maceration | Oral |  |
|  |  |  |  |  | Bronchitis | Brewing | Oral | 50.0 |
|  |  |  |  |  |  | Fermentation | Oral |  |
|  |  |  |  |  |  | Maceration | Oral |  |
| *Commelina communis* L. | KH5068 | Commelinaceae | Daluijangpul | Whole plant | Glycosuria | Decoction | Oral | 100.0 |
| *Cucurbita moschata* | KH5069 | Cucurbitaceae | Hobak | Fruit | Enterotoxin | Warm up in a double | Oral | 100.0 |
| Duchesne |  |  |  |  |  | boiler |  |  |
| *Cudrania tricuspidata* | KH5070 | Moraceae | Kkujippongnamu | Root | Cancer | Decoction | Oral | 50.0 |
| Bureau ex Lavallée |  |  |  | Fruit | Glycosuria | Brewing | Oral | 50.0 |
| *Cynanchum wilfordii* | KH5071 | Apocynaceae | Keunjorong | Root | Knee pain | Brewing | Oral | 33.3 |
| (Maxim.) Hemsl. |  |  |  |  |  | Decoction | Oral |  |
|  |  |  |  |  | Lumbago | Brewing | Oral | 33.3 |
|  |  |  |  |  |  | Decoction | Oral |  |
|  |  |  |  |  | Sthenia | Brewing | Oral | 33.3 |
|  |  |  |  |  |  | Decoction | Oral |  |
| *Dendranthema boreale* | KH5072 | Asteraceae | Sanguk | Flower | Common cold | Decoction | Oral | 50.0 |
| (Makino) Ling ex Kitam. |  |  |  |  | Headache | Decoction | Oral | 50.0 |
| *Dendranthema indicum* (L.) | KH5073 | Asteraceae | Gamguk | Root | Leg pain | Decoction | Oral | 33.3 |
| Des Moul. |  |  |  |  | Neuralgia | Decoction | Oral | 33.3 |
|  |  |  |  |  | Paralysis | Decoction | Oral | 33.3 |
|  |  |  |  | Whole plant | Leg pain | Decoction | Oral |  |
|  |  |  |  |  | Neuralgia | Decoction | Oral |  |
|  |  |  |  |  | Paralysis | Decoction | Oral |  |
| *Dioscorea batatas* Decne. | KH5074 | Dioscoreaceae | Ma | Root | Diarrhea | Infusion | Oral | 100.0 |
|  |  |  |  |  |  | Raw | Oral |  |
| *Diospyros kaki* Thunb. | KH5075 | Ebenaceae | Gamnamu | Fruit | Diarrhea | Raw | Oral | 50.0 |
|  |  |  |  | Leaf | Hypertension | Decoction | Oral | 50.0 |
| *Duchesnea indica* | KH5076 | Rosaceae | Baemttalgi | Fruit | Alopecia | Rubbing | Topical | 28.6 |
| (Andrews) Focke |  |  |  | Whole plant | Skin disease | Decoction | Topical | 71.4 |
|  |  |  |  |  |  | Infusion, wash | Topical |  |
| *Ecklonia cava* Kjellman | KH5077 | Alariaceae | Gamtae | Thallus | Knee pain | Maceration, paste | Topical | 100.0 |
| *Eleutherococcus* | KH5078 | Araliaceae | Ogalpinamu | Bar | Cancer | Decoction | Oral | 6.7 |
| *sessiliflorus* (Rupr. & |  |  |  |  | Panacea | Decoction | Oral | 6.7 |
| Maxim.) S. Y . Hu |  |  |  | Fruit | Neuralgia | Brewing | Oral | 40.0 |
|  |  |  |  |  |  | Decoction | Oral |  |
|  |  |  |  |  |  | Extraction | Oral |  |
|  |  |  |  | Root | Glycosuria | Decoction | Oral | 3.3 |
|  |  |  |  |  | Leg pain | Decoction | Oral | 16.7 |
|  |  |  |  | Stem | Bone disease | Decoction | Oral | 6.7 |
|  |  |  |  |  | Common cold | Tea | Oral | 3.3 |
|  |  |  |  |  | Headache | Decoction | Oral | 6.7 |
|  |  |  |  |  | Leg pain | Decoction | Oral |  |
|  |  |  |  |  | Lumbago | Decoction | Oral | 3.3 |
|  |  |  |  |  | Neuralgia | Decoction | Oral |  |
|  |  |  |  |  | Tonic | Decoction | Oral | 6.7 |
| *Eriobotrya japonica* | KH5079 | Rosaceae | Bipanamu | Leaf | Cancer | Decoction | Oral | 100.0 |
| (Thunb.) Lindl. |  |  |  |  |  |  |  |  |
| *Euonymus alatus* (Thunb.) | KH5080 | Celastraceae | Hwasalnamu | Stem | Cancer | Infusion | Oral | 100.0 |
| Siebold |  |  |  |  |  |  |  |  |
| *Euphorbia humifusa* Willd. | KH5081 | Euphorbiaceae | Ttangbindae | Whole plant | Cancer | Tea | Oral | 100.0 |
| *Euscaphis japonica* (Thunb.) | KH5082 | Staphyleaceae | Malojumttae | Stem | Cancer | Decoction | Oral | 100.0 |
| Kanitz |  |  |  |  |  |  |  |  |
| *Fagopyrum esculentum* | KH5083 | Polygonaceae | Memil | Seed | Appetizer | Porridge | Oral | 5.9 |
| Moench |  |  |  |  | Blood circulation | Powder | Oral | 2.0 |
|  |  |  |  |  | Common cold | Porridge | Oral | 5.9 |
|  |  |  |  |  | Glycosuria | Maceration | Oral | 2.0 |
|  |  |  |  |  | Hypertension | Powder | Oral | 9.8 |
|  |  |  |  |  | Postpartum care | Buckwheat pancakes | Oral | 64.7 |
|  |  |  |  |  |  | Clear soup with | Oral |  |
|  |  |  |  |  |  | dumplings |  |  |
|  |  |  |  |  |  | Porridge | Oral |  |
|  |  |  |  |  |  | Powder | Oral |  |
|  |  |  |  |  | Tonsillitis | Powder | Oral | 2.0 |
|  |  |  |  | Seed coat | Headache | Stuffing of a pillow | Topical | 3.9 |
|  |  |  |  |  | Hypertension | Stuffing of a pillow | Topical |  |
|  |  |  |  |  | Otalgia | Stuffing of a pillow | Topical | 3.9 |
| *Fallopia multiflora* (Thunb.) | KH5084 | Polygonaceae | Hasuo | Root | Cancer | Brewing | Oral | 20.0 |
| Haraldson |  |  |  |  | Hemorrhoid | Brewing | Oral | 20.0 |
|  |  |  |  |  |  | Decoction | Oral |  |
|  |  |  |  |  | Knee pain | Brewing | Oral | 20.0 |
|  |  |  |  |  |  | Decoction | Oral |  |
|  |  |  |  |  | Lumbago | Brewing | Oral | 20.0 |
|  |  |  |  |  |  | Decoction | Oral |  |
|  |  |  |  |  | Sthenia | Brewing | Oral | 20.0 |
|  |  |  |  |  |  | Decoction | Oral |  |
| *Gardenia jasminoides* J.Ellis | KH5085 | Rubiaceae | Chijanamu | Fruit | Bruise | Dough, paste | Topical | 40.0 |
|  |  |  |  |  | Extravasated blood | Dough, paste | Topical | 10.0 |
|  |  |  |  |  | Sprain | Dough, paste | Topical | 50.0 |
| *Gelidium amansii* J. V. | KH5086 | Gelidiaceae | Umutgasari | Thallus | Indigestion | Decoction | Oral | 100.0 |
| Lamour. |  |  |  |  |  |  |  |  |
| *Geranium thunbergii* Siebold | KH5087 | Geraniaceae | Ijilpul | Whole plant | Abdominal pain | Decoction | Oral | 50.0 |
| & Zucc. |  |  |  |  | Diarrhea | Decoction | Oral | 50.0 |
| *Glycine max* (L.) Merr. | KH5088 | Fabaceae | Kong | Leaf | Centipede bite | Rubbing, paste | Topical | 5.0 |
|  |  |  |  | Seed | Common cold | Decoction | Oral | 45.0 |
|  |  |  |  |  | Dog bite | Fermentation, paste | Topical | 10.0 |
|  |  |  |  |  | Head wound | Fermentation, paste | Topical | 15.0 |
|  |  |  |  |  | Skin disease | Maceration, paste | Topical | 5.0 |
|  |  |  |  | Seedling | Cold and flu | Decoction | Oral | 10.0 |
|  |  |  |  |  | Common cold | Decoction | Oral |  |
|  |  |  |  |  | Cough | Decoction | Oral | 10.0 |
| *Glycyrrhiza uralensis* Fisch. | KH5089 | Fabaceae | Gamcho | Root | Facial nerve | Decoction | Oral | 11.1 |
|  |  |  |  |  | paralysis |  |  |  |
|  |  |  |  |  | Gastroenteric | Decoction | Oral | 11.1 |
|  |  |  |  |  | disorder |  |  |  |
|  |  |  |  |  | Sexual enhancement | Boiling | Oral | 11.1 |
|  |  |  |  |  | Spontaneous | Decoction | Oral | 11.1 |
|  |  |  |  |  | abortion |  |  |  |
|  |  |  |  |  | Sterility | Decoction | Oral | 11.1 |
|  |  |  |  |  | Tonsillitis | Powder | Oral | 33.3 |
|  |  |  |  |  | Woman disease | Decoction | Oral | 11.1 |
| *Gossypium indicum* Lam. | KH5090 | Malvaceae | Mokhwa | Seed | Boil | Maceration, paste | Topical | 100.0 |
| *Hibiscus hamabo* Siebold & | KH5091 | Malvaceae | Hwanggeun | Root | Diarrhea | Decoction | Oral | 100.0 |
| Zucc. |  |  |  |  |  |  |  |  |
| *Hizikia fusiformis* (Harvey) | KH5092 | Sargassaceae | Tot | Thallus | Glycosuria | Infusion | Oral | 33.3 |
| Okamura |  |  |  |  | Hypertension | Infusion | Oral | 66.7 |
|  |  |  |  |  |  | Powder | Oral |  |
| *Hordeum vulgare* var. | KH5093 | Poaceae | Bori | Seed | Common cold | Decoction | Oral | 44.4 |
| *hexastichon* (L.) Asch. |  |  |  |  | Indigestion | Fermentation | Oral | 22.2 |
|  |  |  |  |  | Neuralgia | Taffy | Oral | 16.7 |
|  |  |  |  |  | Woman disease | Poultice | Topical | 11.1 |
|  |  |  |  | Seedling | Tonic | Taffy | Oral | 5.6 |
| *Houttuynia cordata* Thunb. | KH5094 | Saururaceae | Yakmomil | Whole part | Gastroenteric | Decoction | Oral | 33.3 |
|  |  |  |  |  | disorder |  |  |  |
|  |  |  |  |  | Liver disease | Decoction | Oral | 66.7 |
|  |  |  |  |  |  | Infusion | Oral |  |
| *Hovenia dulcis* Thunb. | KH5095 | Rhamnaceae | Heotgaenamu | Fruit | Hangover | Decoction | Oral | 10.5 |
|  |  |  |  |  | Liver disease | Decoction | Oral | 36.8 |
|  |  |  |  | Leaf | Hangover | Decoction | Oral |  |
|  |  |  |  | Stem | Bone disease | Decoction | Oral | 10.5 |
|  |  |  |  |  | Cancer | Decoction | Oral | 10.5 |
|  |  |  |  |  | Headache | Decoction | Oral | 10.5 |
|  |  |  |  |  | Liver disease | Tea | Oral |  |
|  |  |  |  |  | Panacea | Decoction | Oral | 10.5 |
|  |  |  |  |  | Tonic | Decoction | Oral | 10.5 |
| *Humulus japonicus* Sieboid | KH5096 | Cannabaceae | Hwansamdeonggul | Whole part | Glycosuria | Decoction | Oral | 50.0 |
| & Zucc. |  |  |  |  | Hypertension | Decoction | Oral | 50.0 |
| *Impatiens balsamina* L. | KH5097 | Balsaminaceae | Bongseonhwa | Root | Irregular | Decoction | Oral | 20.0 |
|  |  |  |  |  | menstruation |  |  |  |
|  |  |  |  |  | Lumbago | Decoction | Oral | 20.0 |
|  |  |  |  |  | Neuralgia | Decoction | Oral | 20.0 |
|  |  |  |  |  | Spontaneous | Decoction | Oral | 20.0 |
|  |  |  |  |  | abortion |  |  |  |
|  |  |  |  |  | Sterility | Decoction | Oral | 20.0 |
| *Imperata cylindrica* var. | KH5098 | Poaceae | Tti | Rhizome | Common cold | Decoction | Oral | 5.6 |
| *koenigii* (Benth.) Druce |  |  |  |  | Hypertension | Decoction | Oral | 5.6 |
|  |  |  |  | Shoot | Snakebite | Maceration, paste | Topical | 88.9 |
| *Kalopanax septemlobus* | KH5099 | Araliaceae | Eumnamu | Stem | Arthritis | Decoction | Oral | 25.0 |
| (Thunb.) Koidz. |  |  |  |  | Blood circulation | Infusion | Oral | 16.7 |
|  |  |  |  |  | disorder |  |  |  |
|  |  |  |  |  | Bone disease | Decoction | Oral | 16.7 |
|  |  |  |  |  | Headache | Decoction | Oral | 16.7 |
|  |  |  |  |  | Neuralgia | Decoction | Oral | 8.3 |
|  |  |  |  |  | Tonic | Decoction | Oral | 16.7 |
| *Lactuca sativa* L. | KH5100 | Asteraceae | Sangchu | Leaf | Snakebite | Maceration, paste | Topical | 100.0 |
| *Lagenaria leucantha* Rusby | KH5101 | Cucurbitaceae | Bak | Inside of | Common cold | Decoction | Oral | 100.0 |
|  |  |  |  | fruit |  |  |  |  |
|  |  |  |  | Seed | Common cold | Decoction | Oral |  |
| *Ledebouriella seseloides* | KH5102 | Apiaceae | Bangpung | Root | Common cold | Decoction | Oral | 28.6 |
| H. Wolff |  |  |  |  | Paralysis | Decoction | Oral | 42.9 |
|  |  |  |  |  | Tonic | Decoction | Oral | 28.6 |
| *Lentinula edodes* (Berk.) | KH5103 | Pleurotaceae | Pyogo | Carpophore | Cancer | Decoction | Oral | 100.0 |
| Sing. |  |  |  |  |  |  |  |  |
| *Leonurus japonicus* Houtt. | KH5104 | Lamiaceae | Ikmocho | Aerial part | Abdominal pain | Decoction | Oral | 17.9 |
|  |  |  |  |  |  | Juice | Oral |  |
|  |  |  |  |  |  | Pill | Oral |  |
|  |  |  |  |  |  | Taffy | Oral |  |
|  |  |  |  |  | Bloody discharge | Decoction | Oral | 2.6 |
|  |  |  |  |  | Common cold | Decoction | Oral | 1.3 |
|  |  |  |  |  | Dysmenorrhea | Decoction | Oral | 3.8 |
|  |  |  |  |  | Gastroenteric | Decoction | Oral | 5.1 |
|  |  |  |  |  | disorder |  |  |  |
|  |  |  |  |  | Irregular | Decoction | Oral | 34.6 |
|  |  |  |  |  | menstruation | Infusion | Oral |  |
|  |  |  |  |  |  | Taffy | Oral |  |
|  |  |  |  |  | Postpartum care | Decoction | Oral | 3.8 |
|  |  |  |  |  | Spontaneous | Decoction | Oral | 1.3 |
|  |  |  |  |  | abortion |  |  |  |
|  |  |  |  |  | Sterility | Decoction | Oral | 6.4 |
|  |  |  |  |  | Woman disease | Decoction | Oral | 23.1 |
|  |  |  |  |  |  | Taffy | Oral |  |
| *Liriope platyphylla* F. T. | KH5105 | Convallariaceae | Maekmundong | Root | Asthma | Decoction | Oral | 50.0 |
| Wang & Tang |  |  |  |  | Cough | Decoction | Oral | 50.0 |
| *Lithospermum erythrorhizon* | KH5106 | Boraginaceae | Jichi | Root | Obesity | Brewing | Oral | 100.0 |
| Siebold & Zucc. |  |  |  |  |  | Decoction | Oral |  |
| *Lonicera japonica* Thunb. | KH5107 | Caprifoliaceae | Indongdeonggul | Flower | Chronic cough | Decoction | Oral | 8.3 |
|  |  |  |  |  |  | Extraction | Oral |  |
|  |  |  |  |  | Common cold | Brewing | Oral | 79.2 |
|  |  |  |  |  |  | Decoction | Oral |  |
|  |  |  |  |  | Cough | Decoction | Oral | 1.4 |
|  |  |  |  |  | Gastroenteric | Decoction | Oral | 1.4 |
|  |  |  |  |  | disorder |  |  |  |
|  |  |  |  | Leaf | Hypertension | Extraction | Oral | 2.8 |
|  |  |  |  | Stem | Atopic dermatitis | Infusion | Topical | 6.9 |
|  |  |  |  |  | Common cold | Decoction | Oral |  |
|  |  |  |  |  | Hypertension | Extraction | Oral |  |
| *Luffa cylindrica* M. Roem. | KH5108 | Cucurbitaceae | Susemioi | Fruit | Asthma | Decoction | Oral | 33.3 |
|  |  |  |  |  | Bronchitis | Decoction | Oral | 50.0 |
|  |  |  |  |  | Common cold | Decoction | Oral | 16.7 |
|  |  |  |  | Sap | Asthma | Sap | Oral |  |
| *Lycium chinense* Mill. | KH5109 | Solanaceae | Gugijanamu | Fruit | Gastroenteric | Decoction | Oral | 33.3 |
|  |  |  |  |  | disorder |  |  |  |
|  |  |  |  |  | Neuralgia | Decoction | Oral | 33.3 |
|  |  |  |  |  |  | Tea | Oral |  |
|  |  |  |  |  | Tonic | Brewing | Oral | 33.3 |
| *Lycoris radiata* (L'Her.) | KH5110 | Amaryllidaceae | Seoksan | Root | Cough | Decoction | Oral | 50.0 |
| Herb. |  |  |  |  | Sputum | Decoction | Oral | 50.0 |
| *Machilus thunbergii* Siebold | KH5111 | Lauraceae | Hubaknamu | Bark | Cancer | Decoction | Oral | 25.0 |
| & Zucc. |  |  |  |  | Gastroenteric | Decoction | Oral | 75.0 |
|  |  |  |  |  | disorder |  |  |  |
| *Melia azedarach* L. | KH5112 | Meliaceae | Meolguseulnamu | Bark | Induced abortion | Juice | Oral | 20.7 |
|  |  |  |  | Fruit | Abdominal pain | Decoction | Oral | 31.0 |
|  |  |  |  |  | Parasite | Taffy | Oral | 17.2 |
|  |  |  |  | Leaf | Parasite | Decoction | Oral |  |
|  |  |  |  | Root | Constipation | Decoction | Oral | 13.8 |
|  |  |  |  |  | Induced abortion | Juice | Oral |  |
|  |  |  |  | Root bark | Gastroenteric | Taffy | Oral | 3.4 |
|  |  |  |  |  | disorder |  |  |  |
|  |  |  |  |  | Parasite | Taffy | Oral |  |
|  |  |  |  | Stem | Pleurodynia | Decoction | Oral | 13.8 |
| *Mentha arvensis* var. | KH5113 | Lamiaceae | Bakha | Leaf | Abdominal pain | Juice | Oral | 25.0 |
| *piperascens* Malv. ex |  |  |  |  | Common cold | Boiling | Oral | 75.0 |
| Holmes |  |  |  |  |  | Tea | Oral |  |
| *Morus alba* L. | KH5114 | Moraceae | Ppongnamu | Fruit | Asthma | Brewing | Oral | 15.0 |
|  |  |  |  |  |  | Extraction | Oral |  |
|  |  |  |  |  |  | Raw | Oral |  |
|  |  |  |  |  | Cough | Brewing | Oral | 15.0 |
|  |  |  |  |  |  | Extraction | Oral |  |
|  |  |  |  |  |  | Raw | Oral |  |
|  |  |  |  |  | Glycosuria | Brewing | Oral | 25.0 |
|  |  |  |  |  |  | Extraction | Oral |  |
|  |  |  |  |  |  | Raw | Oral |  |
|  |  |  |  |  | Lung disease | Brewing | Oral | 15.0 |
|  |  |  |  |  |  | Extraction | Oral |  |
|  |  |  |  |  |  | Raw | Oral |  |
|  |  |  |  | Leaf | Glycosuria | Decoction | Oral |  |
|  |  |  |  | Root | Cancer | Decoction | Oral | 20.0 |
|  |  |  |  |  | Panacea | Decoction | Oral | 10.0 |
|  |  |  |  | Stem | Glycosuria | Decoction | Oral |  |
| *Mosla japonica* (Oliv.) | KH5115 | Lamiaceae | Sandeulkkae | Leaf | Common cold | Decoction | Oral | 100.0 |
| Maxim. |  |  |  | Stem | Common cold | Decoction | Oral |  |
| *Musa basjoo* Siebold & | KH5116 | Musaceae | Pacho | Root | Woman disease | Juice | Oral | 100.0 |
| Zucc. ex Iinuma |  |  |  |  |  |  |  |  |
| *Neolitsea sericea* (Blume) | KH5117 | Lauraceae | Chamsiknamu | Fruit | Cradle cap | Oil | Topical | 100.0 |
| Koidz. |  |  |  |  |  |  |  |  |
| *Nicotiana tabacum* L. | KH5118 | Solanaceae | Dambae | Leaf | Bruise | Paste | Topical | 38.5 |
|  |  |  |  |  | Hemostasis | Paste | Topical | 61.5 |
|  |  |  |  |  |  | Rubbing, paste | Topical |  |
| *Oenanthe javanica* DC. | KH5119 | Apiaceae | Minari | Aerial part | Hangover | Juice | Oral | 13.3 |
|  |  |  |  |  | Lymphnoditis | Maceration, paste | Topical | 6.7 |
|  |  |  |  |  | Pesticide | Juice | Oral | 53.3 |
|  |  |  |  |  | detoxification | Raw | Oral |  |
|  |  |  |  |  | Pus | Maceration, paste | Topical | 26.7 |
| *Opuntia ficus-indica* var. | KH5120 | Cactaceae | Sonbadakseoninjang | Stem | Bruise | Maceration, paste | Topical | 25.9 |
| *saboten* Makino |  |  |  |  | Burn | Paste | Topical | 29.6 |
|  |  |  |  |  | Chronic myofascial | Paste | Topical | 14.8 |
|  |  |  |  |  | pain |  |  |  |
|  |  |  |  |  | Constipation | Raw | Oral | 3.7 |
|  |  |  |  |  | Gastroenteric | Raw | Oral | 7.4 |
|  |  |  |  |  | disorder |  |  |  |
|  |  |  |  |  | Knee pain | Paste | Topical | 7.4 |
|  |  |  |  |  | Physical pain | Maceration, paste | Topical | 7.4 |
|  |  |  |  |  | Skin disease | Paste | Topical | 3.7 |
| *Oryza sativa* var*. terrestis* | KH5121 | Poaceae | Sandu | Seed | Abdominal pain | Porridge | Oral | 11.1 |
| Makino |  |  |  |  | Appetizer | Porridge | Oral | 8.3 |
|  |  |  |  |  | Diarrhea | Porridge | Oral | 55.6 |
|  |  |  |  |  | Herpes labialis | Boiling | Topical | 2.8 |
|  |  |  |  |  | Lumbago | Porridge | Oral | 8.3 |
|  |  |  |  |  | Stomach | Porridge | Oral | 5.6 |
|  |  |  |  |  | problem |  |  |  |
|  |  |  |  |  | Tonic | Porridge | Oral | 8.3 |
| *Paeonia japonica* (Makino) | KH5122 | Paeoniaceae | Baekjakyak | Root | Analgesic | Brewing | Oral | 100.0 |
| Miyabe & Takeda |  |  |  |  |  | Decoction | Oral |  |
| *Panax ginseng* C. A. Mey. | KH5123 | Araliaceae | Insam | Root | Tonic | Porridge | Oral | 100.0 |
| *Papaver somniferum* L. | KH5124 | Papaveraceae | Yanggwibi | Fruit | Furuncle | Decoction | Topical | 80.0 |
|  |  |  |  | Latex | Boil | Extraction, paste | Topical | 6.7 |
|  |  |  |  |  | Furuncle | Raw, applying | Topical | 80.0 |
|  |  |  |  |  | Gastroenteric | Extraction | Oral | 6.7 |
|  |  |  |  |  | disorder |  |  |  |
|  |  |  |  |  | Panacea | Extraction | Oral | 6.7 |
|  |  |  |  | Stem | Furuncle | Decoction | Topical | 80.0 |
| *Perilla frutescens* (L.) Britton | KH5125 | Lamiaceae | Soyeop | Leaf | Common cold | Decoction | Oral | 100.0 |
| *Petasites rubellus* (J. F. | KH5126 | Asteraceae | Gaemeowi | Root | Tonsillitis | Brewing | Oral | 100.0 |
| Gmel.) Toman |  |  |  |  |  |  |  |  |
| *Peucedanum japonicum* | KH5127 | Apiaceae | Gaetgireumnamul | Aerial part | Paralysis | Infusion | Oral | 33.3 |
| Thunb. |  |  |  | Fruit | Headache | Stuffing of a pillow | Topical | 13.3 |
|  |  |  |  | Root | Dental pain | Dissolution | Topical | 26.7 |
|  |  |  |  |  | Paralysis | Decoction | Oral |  |
|  |  |  |  |  | Postpartum care | Decoction | Oral | 26.7 |
|  |  |  |  | Stem | Paralysis | Decoction | Oral |  |
| *Phellodendron amurense* | KH5128 | Rutaceae | Hwangbyeoknamu | Bark | Abdominal pain | Brewing | Oral | 9.5 |
| Rupr. |  |  |  |  |  | Decoction | Oral |  |
|  |  |  |  |  | Bruise | Maceration, paste | Topical | 4.8 |
|  |  |  |  |  | Facial nerve | Decoction | Oral | 4.8 |
|  |  |  |  |  | paralysis |  |  |  |
|  |  |  |  | Inner layer | Gastroenteric | Decoction | Oral | 19.0 |
|  |  |  |  | of bark | disorder |  |  |  |
|  |  |  |  |  | Stomatitis | Paste | Topical | 57.1 |
|  |  |  |  | Bark | Tonic | Brewing | Oral | 4.8 |
| *Phryma leptostachya* var. | KH5129 | Phrymaceae | Paripul | Root | Acne | Infusion, applying | Topical | 1.7 |
| *oblongifolia* (Koidz.) |  |  |  |  | Bone disease | Taffy | Oral | 3.4 |
| Honda |  |  |  |  | Cancer | Decoction | Oral |  |
|  |  |  |  |  |  | Infusion | Oral | 8.6 |
|  |  |  |  |  | Gout | Decoction | Oral | 1.7 |
|  |  |  |  |  | Pruritus | Brewing, paste | Topical | 19.0 |
|  |  |  |  |  |  | Maceration, paste | Topical | 19.0 |
|  |  |  |  |  | Skin disease | Brewing, applying | Topical | 62.1 |
|  |  |  |  |  |  | Decoction | Topical |  |
|  |  |  |  |  |  | Infusion | Topical |  |
|  |  |  |  |  |  | Infusion | Oral |  |
|  |  |  |  |  |  | Maceration, paste | Topical |  |
|  |  |  |  |  |  | Preserve in salt, paste | Topical |  |
|  |  |  |  | Aerial part | Woman disease | Decoction | Oral | 3.4 |
| *Phyllostachys bambusoides* | KH5130 | Poaceae | Wangdae | Shoot | Common cold | Decoction | Oral | 100.0 |
| Siebold & Zucc. |  |  |  | Stem | Common cold | Decoction | Oral |  |
| *Pinellia ternata* Ten. ex | KH5131 | Araceae | Banha | Root | Sputum | Decoction | Oral | 100.0 |
| Breitenb. |  |  |  |  |  |  |  |  |
| *Pinus densiflora* Siebold & | KH5132 | Pinaceae | Sonamu | Bark | Frostbite | Paste | Topical | 2.2 |
| Zucc. |  |  |  | Leaf | Blood circulation | Extraction | Oral | 4.3 |
|  |  |  |  |  | Hypertension | Brewing | Oral | 10.9 |
|  |  |  |  |  |  | Tea | Oral |  |
|  |  |  |  |  | Knee pain | Poultice | Topical | 2.2 |
|  |  |  |  |  | Lumbago | Boiling | Oral | 13.0 |
|  |  |  |  |  | Paralysis | Brewing | Oral | 8.7 |
|  |  |  |  |  |  | Tea | Oral |  |
|  |  |  |  | Pine cone | Eczema | Decoction | Topical | 2.2 |
|  |  |  |  |  | Scrotal edema | Decoction | Topical | 2.2 |
|  |  |  |  | Resin | Cradle cap | Paste | Topical | 6.5 |
|  |  |  |  |  | Dental pain | Paste | Topical | 2.2 |
|  |  |  |  |  | Pus | Paste | Topical | 8.7 |
|  |  |  |  |  | Skin disease | Salve, paste | Topical | 8.7 |
|  |  |  |  | Shoot | Abrasion | Brewing | Oral | 4.3 |
|  |  |  |  |  | Bruise | Brewing | Oral | 4.3 |
|  |  |  |  | Sprout | Common cold | Extraction | Oral | 8.7 |
|  |  |  |  |  |  | Maceration | Oral |  |
|  |  |  |  |  | Lumbago | Brewing | Oral |  |
|  |  |  |  |  | Tonic | Brewing | Oral | 8.7 |
|  |  |  |  | Young leaf | Glycosuria | Extraction | Oral | 2.2 |
|  |  |  |  |  | Hypertension | Extraction | Oral |  |
| *Plantago asiatica* L. | KH5133 | Plantaginaceae | Jilgyeongi | Leaf | Abrasion | Maceration, paste | Topical | 4.1 |
|  |  |  |  |  | Bruise | Maceration, paste | Topical | 4.1 |
|  |  |  |  |  | Centipede bite | Maceration, paste | Topical | 4.1 |
|  |  |  |  |  | Edema | Roast, paste | Topical | 5.2 |
|  |  |  |  |  | Gastroenteric | Decoction | Oral | 9.3 |
|  |  |  |  |  | disorder |  |  |  |
|  |  |  |  |  | Hemostasis | Rubbing, paste | Topical | 2.1 |
|  |  |  |  |  | Tingling | Maceration, paste | Topical | 4.1 |
|  |  |  |  | Petiole | Gastroenteric | Decoction | Oral |  |
|  |  |  |  |  | disorder |  |  |  |
|  |  |  |  | Whole plant | Anuresis | Decoction | Oral | 9.3 |
|  |  |  |  |  | Centipede bite | Maceration, paste | Topical |  |
|  |  |  |  |  | Cystitis | Decoction | Oral | 8.2 |
|  |  |  |  |  | Diarrhea | Decoction | Oral | 2.1 |
|  |  |  |  |  | Diuresis | Decoction | Oral | 5.2 |
|  |  |  |  |  |  | Infusion | Oral |  |
|  |  |  |  |  | Edema | Decoction | Oral |  |
|  |  |  |  |  | Gastroenteric | Decoction | Oral |  |
|  |  |  |  |  | disorder |  |  |  |
|  |  |  |  |  | Obesity | Decoction | Oral | 4.1 |
|  |  |  |  |  | Pollakiuria | Decoction | Oral | 20.6 |
|  |  |  |  |  | Prostate disease | Decoction | Oral | 5.2 |
|  |  |  |  |  | Renal disease | Decoction | Oral | 5.2 |
|  |  |  |  |  | Woman disease | Decoction | Oral | 7.2 |
| *Platycodon grandiflorus* | KH5134 | Campanulaceae | Doraji | Root | Cough | Brewing | Oral | 75.0 |
| A. DC. |  |  |  |  |  | Decoction | Oral |  |
|  |  |  |  |  |  | Extraction | Oral |  |
|  |  |  |  |  | Sputum | Maceration | Oral | 25.0 |
| *Platycodon grandiflorus* for. | KH5135 | Campanulaceae | Baekdoraji | Root | Postpartum care | Infusion | Oral | 60.0 |
| *albiflorus* (Honda) H. Hara |  |  |  |  | Spontaneous | Decoction | Oral | 20.0 |
|  |  |  |  |  | abortion |  |  |  |
|  |  |  |  |  | Sterility | Decoction | Oral | 20.0 |
| *Polygonatum falcatum* A. | KH5136 | Convallariaceae | Jinhwangjeong | Rhizome | Neuralgia | Decoction | Oral | 100.0 |
| Gray |  |  |  |  |  |  |  |  |
| *Polygonatum odoratum* | KH5137 | Convallariaceae | Dunggulre | Rhizome | Gastroenteric | Tea | Oral | 50.0 |
| var. *pluriflorum* (Miq.) |  |  |  |  | disorder |  |  |  |
| Ohwi |  |  |  |  | Glycosuria | Tea | Oral | 50.0 |
| *Polygonum aviculare* L. | KH5138 | Polygonaceae | Madipul | Aerial part | Skin disease | Infusion | Topical | 11.8 |
|  |  |  |  | Root | Arthritis | Decoction | Oral | 23.5 |
|  |  |  |  |  | Bone disease | Decoction | Oral | 17.6 |
|  |  |  |  |  | Knee pain | Decoction | Oral | 23.5 |
|  |  |  |  |  | Lumbago | Decoction | Oral | 5.9 |
|  |  |  |  | Stem | Glycosuria | Decoction | Oral | 17.6 |
| *Poncirus trifoliata* (L.) Raf. | KH5139 | Rutaceae | Taengjanamu | Fruit | Indigestion | Decoction | Oral | 100.0 |
| *Potentilla chinensis* Ser. | KH5140 | Rosaceae | Ttakjikkot | Leaf | Tingling | Dissolution, | Oral | 100.0 |
| var*. chinensis* |  |  |  |  |  | maceration |  |  |
|  |  |  |  | Root | Tingling | Brewing | Oral |  |
|  |  |  |  |  |  | Dissolution, | Oral |  |
|  |  |  |  |  |  | maceration |  |  |
| *Prunus mume* Siebold & | KH5141 | Rosaceae | Maesilnamu | Fruit | Food poisoning | Extraction | Oral | 50.0 |
| Zucc. |  |  |  |  | Indigestion | Extraction | Oral | 50.0 |
| *Prunus tomentosa* Thunb. | KH5142 | Rosaceae | Aengdonamu | Fruit | Obesity | Brewing | Oral | 100.0 |
|  |  |  |  |  |  | Maceration | Oral |  |
|  |  |  |  |  |  | Raw | Oral |  |
| *Pseudosasa japonica* | KH5143 | Poaceae | Idea | Leaf | Common cold | Decoction | Oral | 100.0 |
| Makino |  |  |  |  |  |  |  |  |
| *Pteridium aquilinum* | KH5144 | Dennstaedtiaceae | Gosari | Young | Snakebite | Maceration, paste | Topical | 100.0 |
| var*. latiusculum* (Desv.) |  |  |  | frond |  |  |  |  |
| Underw. ex A.Hell. |  |  |  |  |  |  |  |  |
| *Pueraria lobata* (Willd.) Ohwi | KH5145 | Fabaceae | Chik | Root | Common cold | Decoction | Oral | 33.3 |
|  |  |  |  |  | Gastroenteric | Brewing | Oral | 66.7 |
|  |  |  |  |  | disorder | Decoction | Oral |  |
| *Pulsatilla koreana* Y. (Yabe | KH5146 | Ranunculaceae | Halmikkot | Leaf | Stomach cramp | Rubbing, smelling | Nasal | 100.0 |
| ex Nakai) T.Mori |  |  |  |  |  |  |  |  |
| *Punica granatum* L. | KH5147 | Lythraceae | Seokryunamu | Fruit | Abdominal pain | Extraction | Oral | 100.0 |
|  |  |  |  |  |  | Infusion | Oral |  |
| *Pyrus pyrifolia* Nakai | KH5148 | Rosaceae | Baenamu | Fruit | Common cold | Decoction | Oral | 82.9 |
|  |  |  |  |  |  | Simmer | Oral |  |
|  |  |  |  |  |  | Tea | Oral |  |
|  |  |  |  |  | Cough | Decoction | Oral | 17.1 |
| *Raphanus sativus* L. | KH5149 | Brassicaceae | Mu | Root | Common cold | Decoction | Oral | 87.5 |
|  |  |  |  |  | Cough | Decoction | Oral | 12.5 |
| *Rehmannia glutinosa* | KH5150 | Scrophulariaceae | Jihwang | Root | Bruise | Maceration, paste | Topical | 14.3 |
| Steud. |  |  |  |  | Cancer | Decoction | Oral | 14.3 |
|  |  |  |  |  | Edema | Maceration, paste | Topical | 14.3 |
|  |  |  |  |  | Extravasated blood | Dough, paste | Topical | 7.1 |
|  |  |  |  |  | Neuralgia | Brewing | Oral | 7.1 |
|  |  |  |  |  | Panacea | Brewing | Oral | 21.4 |
|  |  |  |  |  |  | Decoction | Oral |  |
|  |  |  |  |  | Sprain | Maceration, paste | Topical | 14.3 |
|  |  |  |  |  | Tonic | Brewing | Oral | 7.1 |
| *Rheum rhabarbarum* L. | KH5151 | Polygonaceae | Daehwang | Root | Diarrhea | Decoction | Oral | 100.0 |
| *Rhus verniciflua* Stokes | KH5152 | Anacardiaceae | Otnamu | Stem | Gastroenteric | Infusion | Oral | 44.4 |
|  |  |  |  |  | disorder |  |  |  |
|  |  |  |  |  | Raynaud's | Decoction | Oral | 27.8 |
|  |  |  |  |  | phenomenon |  |  |  |
|  |  |  |  |  | Tonic | Infusion | Oral | 27.8 |
| *Rhynchosia volubilis* Lour. | KH5153 | Fabaceae | Yeoukong | Seed | Common cold | Decoction | Oral | 100.0 |
| *Ricinus communis* L. | KH5154 | Euphorbiaceae | Pimaja | Leaf | Furuncle | Paste | Topical | 4.9 |
|  |  |  |  |  | Skin disease | Beating, Paste | Topical | 9.8 |
|  |  |  |  | Seed | Abdominal pain | Oil | Oral | 9.8 |
|  |  |  |  |  | Burn | Oil | Topical | 4.9 |
|  |  |  |  |  | Constipation | Oil | Oral | 70.7 |
| *Rosa multiflora* Thunb. | KH5155 | Rosaceae | Jjilrekkot | Root | Leg pain | Decoction | Oral | 50.0 |
| var. *multiflora* |  |  |  |  | Neuralgia | Decoction | Oral | 50.0 |
| *Rubus coreanus* Miq. | KH5156 | Rosaceae | Bokbunjattalgi | Fruit | Sexual enhancement | Brewing | Oral | 100.0 |
| *Rubus crataegifolius* Bunge | KH5157 | Rosaceae | Santtalgi | Fruit | Bone disease | Brewing | Oral | 50.0 |
|  |  |  |  |  | Sexual enhancement | Extraction | Oral | 25.0 |
|  |  |  |  |  | Tonic | Brewing | Oral | 25.0 |
| *Rubus croceacanthus* | KH5158 | Rosaceae | Geomeunttalgi | Fruit | Panacea | Brewing | Oral | 100.0 |
| H.Lev. |  |  |  |  |  |  |  |  |
| *Rubus hirsutus* Thunb. | KH5159 | Rosaceae | Jangttalgi | Fruit | Sexual enhancement | Brewing | Oral | 100.0 |
| *Rubus parvifolius* L. | KH5160 | Rosaceae | Meongseokttalgi | Fruit | Common cold | Brewing | Oral | 50.0 |
|  |  |  |  |  | Neuralgia | Brewing | Oral | 50.0 |
| *Rumex acetosa* L. | KH5161 | Polygonaceae | Suyeong | Root | Diarrhea | Decoction | Oral | 100.0 |
| *Sageretia theezans* (L.) | KH5162 | Rhamnaceae | Sangdongnamu | Fruit | Leg pain | Brewing | Oral | 62.5 |
| Brongn. |  |  |  |  | Neuralgia | Brewing | Oral | 37.5 |
| *Salix babylonica* L. | KH5163 | Salicaceae | Suyangbeodeul | Stem | Measles | Decoction | Oral | 100.0 |
| *Salix koreensis* Andersson | KH5164 | Salicaceae | Beodeunamu | Bark | Fracture | Binding | Topical | 100.0 |
| *Sanguisorba officinalis* L. | KH5165 | Rosaceae | Oipul | Leaf | Pus | Infusion | Topical | 6.7 |
|  |  |  |  |  | Skin disease | Infusion | Topical | 10.0 |
|  |  |  |  | Root | Abdominal pain | Decoction | Oral | 13.3 |
|  |  |  |  |  | Cancer | Decoction | Oral | 3.3 |
|  |  |  |  |  | Gastroenteric | Decoction | Oral | 6.7 |
|  |  |  |  |  | disorder |  |  |  |
|  |  |  |  |  | Liver disease | Decoction | Oral | 3.3 |
|  |  |  |  |  | Neuralgia | Decoction | Oral | 3.3 |
|  |  |  |  |  | Postpartum care | Decoction | Oral | 13.3 |
|  |  |  |  |  | Pus | Infusion | Topical |  |
|  |  |  |  |  | Raynaud's | Brewing | Oral | 20.0 |
|  |  |  |  |  | phenomenon | Decoction | Oral |  |
|  |  |  |  |  | Skin disease | Infusion | Topical |  |
|  |  |  |  |  | Woman disease | Brewing | Oral | 20.0 |
|  |  |  |  |  |  | Decoction | Oral |  |
| *Saururus chinensis* Hort. ex | KH5166 | Saururaceae | Sambaekcho | Whole part | Cancer | Decoction | Oral | 50.0 |
| Loudon |  |  |  |  | Gastroenteric | Infusion | Oral | 50.0 |
|  |  |  |  |  | disorder |  |  |  |
| *Schisandra chinensis* | KH5167 | Schisandraceae | Omija | Fruit | Bronchitis | Tea | Oral | 18.8 |
| (Turcz.) Baill. |  |  |  |  | Eye strain | Brewing | Oral | 18.8 |
|  |  |  |  |  | Gastroenteric | Brewing | Oral | 18.8 |
|  |  |  |  |  | disorder |  |  |  |
|  |  |  |  |  | Liver disease | Brewing | Oral | 18.8 |
|  |  |  |  |  | Neuralgia | Brewing | Oral | 12.5 |
|  |  |  |  |  |  | Decoction | Oral |  |
|  |  |  |  |  | Tonic | Brewing | Oral | 12.5 |
|  |  |  |  | Root | Bronchitis | Tea | Oral |  |
|  |  |  |  |  | Eye strain | Brewing | Oral |  |
|  |  |  |  |  | Gastroenteric | Brewing | Oral |  |
|  |  |  |  |  | disorder |  |  |  |
|  |  |  |  |  | Liver disease | Brewing | Oral |  |
|  |  |  |  | Stem | Eye strain | Brewing | Oral |  |
|  |  |  |  |  | Gastroenteric | Brewing | Oral |  |
|  |  |  |  |  | disorder |  |  |  |
|  |  |  |  |  | Liver disease | Brewing | Oral |  |
| *Selaginella tamariscina* (P. | KH5168 | Selaginellaceae | Bucheoson | Whole plant | Skin disease | Fumigation | Topical | 100.0 |
| Beauv.) Spring |  |  |  |  |  |  |  |  |
| *Sesamum indicum* L. | KH5169 | Pedaliaceae | Chamkkae | Seed | Pruritus | Oil | Topical | 53.3 |
|  |  |  |  |  | Skin disease | Oil | Topical | 33.3 |
|  |  |  |  |  | Tonic | Oil | Oral | 6.7 |
|  |  |  |  |  | Xerocheilia | Oil | Topical | 6.7 |
| *Setaria italica* (L.) P. Beauv. | KH5170 | Poaceae | Jo | Seed | Indigestion | Fermentation | Oral | 66.7 |
|  |  |  |  |  | Neuralgia | Taffy | Oral | 25.0 |
|  |  |  |  |  | Tonic | Brewing | Oral | 8.3 |
| *Sigesbeckia glabrescens* | KH5171 | Asteraceae | Jindeukchal | Whole plant | Cancer | Decoction | Oral | 33.3 |
| (Makino) Makino |  |  |  |  | Eczema | Infusion | Topical | 33.3 |
|  |  |  |  |  | Pruritus | Infusion | Topical | 33.3 |
| *Solanum nigrum* L. | KH5172 | Solanaceae | Kkamajung | Leaf | Eczema | Maceration, paste | Topical | 100.0 |
| *Solanum tuberosum* L. | KH5173 | Solanaceae | Gamja | Tuber | Bruise | Maceration, paste | Topical | 10.5 |
|  |  |  |  |  | Burn | Maceration, paste | Topical | 89.5 |
| *Sophora flavescens* Aiton | KH5174 | Fabaceae | Gosam | Fruit | Gastroenteric | Raw | Oral | 47.1 |
|  |  |  |  |  | disorder |  |  |  |
|  |  |  |  | Root | Gastroenteric | Decoction | Oral |  |
|  |  |  |  |  | disorder |  |  |  |
|  |  |  |  |  | Knee pain | Poultice | Topical | 23.5 |
|  |  |  |  |  | Lumbago | Poultice | Topical | 23.5 |
|  |  |  |  |  | Neuralgia | Decoction | Oral | 5.9 |
| *Styrax japonicus* Siebold & | KH5175 | Styracaceae | Ttaejuknamu | Bark | Snakebite | Maceration, paste | Topical | 100.0 |
| Zucc. |  |  |  |  |  |  |  |  |
| *Taraxacum platycarpum* | KH5176 | Asteraceae | Mindeulre | Whole plant | Cancer | Decoction | Oral | 16.7 |
| Dahlst. |  |  |  |  |  | Infusion | Oral |  |
|  |  |  |  |  | Gastroenteric | Decoction | Oral | 22.2 |
|  |  |  |  |  | disorder |  |  |  |
|  |  |  |  |  | Glycosuria | Decoction | Oral | 11.1 |
|  |  |  |  |  | Liver disease | Decoction | Oral | 16.7 |
|  |  |  |  |  | Panacea | Infusion | Oral | 22.2 |
|  |  |  |  |  | Pollakiuria | Decoction | Oral | 11.1 |
| *Torreya nucifera* (L.) Siebold | KH5177 | Taxaceae | Bijanamu | Fruit | Parasite | Drying | Oral | 100.0 |
| & Zucc. |  |  |  |  |  | Powder | Oral |  |
|  |  |  |  |  |  | Raw | Oral |  |
| *Trichosanthes kirilowii* | KH5178 | Cucurbitaceae | Haneultari | Fruit | Bruise | Brewing | Oral | 2.1 |
| Maxim. |  |  |  |  | Common cold | Decoction | Oral | 60.4 |
|  |  |  |  |  |  | Infusion | Oral |  |
|  |  |  |  |  | Cough | Decoction | Oral | 8.3 |
|  |  |  |  |  | Lumbago | Brewing | Oral | 8.3 |
|  |  |  |  |  |  | Infusion | Oral |  |
|  |  |  |  | Seed | Common cold | Decoction | Oral |  |
|  |  |  |  | Tuberous | Cancer | Decoction | Oral | 4.2 |
|  |  |  |  | root | Common cold | Decoction | Oral |  |
|  |  |  |  |  | Edema | Maceration, paste | Topical | 4.2 |
|  |  |  |  |  | Neuralgia | Decoction | Oral | 2.1 |
|  |  |  |  |  | Panacea | Decoction | Oral | 4.2 |
|  |  |  |  |  | Sprain | Maceration, paste | Topical | 6.3 |
| *Trichosanthes kirilowii* var. | KH5179 | Cucurbitaceae | Noranghaneultari | Fruit | Bronchitis | Decoction | Oral | 13.3 |
| *japonica* Kitam. |  |  |  |  | Common cold | Brewing | Oral | 63.3 |
|  |  |  |  |  |  | Decoction | Oral |  |
|  |  |  |  |  | Cough | Decoction | Oral | 13.3 |
|  |  |  |  | Sap | Gastroenteric | Sap | Oral | 10.0 |
|  |  |  |  |  | disorder |  |  |  |
|  |  |  |  | Tuberous | Common cold | Decoction | Oral |  |
|  |  |  |  | root |  |  |  |  |
| *Triticum aestivum* L. | KH5180 | Poaceae | Mil | Seed | Abdominal pain | Pill | Oral | 16.7 |
|  |  |  |  |  | Bruise | Clear soup with | Oral | 33.3 |
|  |  |  |  |  |  | dumplings |  |  |
|  |  |  |  |  | Chronic myofascial | Clear soup with | Oral | 14.8 |
|  |  |  |  |  | pain | dumplings |  |  |
|  |  |  |  |  | Extravasated blood | Dough | Topical | 8.3 |
|  |  |  |  |  | Lumbago | Clear soup with | Oral | 8.3 |
|  |  |  |  |  |  | dumplings |  |  |
| *Ulmus davidiana* var*.* | KH5181 | Ulmaceae | Neureupnamu | Bark | Cancer | Decoction | Oral | 9.8 |
| *japonica* (Rehder) Nakai |  |  |  |  | Furuncle | Maceration, paste | Topical | 8.5 |
|  |  |  |  |  | Gastroenteric | Simmer | Oral | 15.9 |
|  |  |  |  |  | disorder |  |  |  |
|  |  |  |  |  | Headache | Maceration, paste | Topical | 2.4 |
|  |  |  |  |  | Liver disease | Decoction | Oral | 4.9 |
|  |  |  |  |  | Nasal obstruction | Decoction | Oral | 1.2 |
|  |  |  |  |  | Panacea | Decoction | Oral | 2.4 |
|  |  |  |  |  | Pus | Maceration, paste | Topical | 18.3 |
|  |  |  |  |  |  | Rolling | Topical |  |
|  |  |  |  |  | Skin disease | Maceration, paste | Topical | 11.0 |
|  |  |  |  | Leaf | Gastroenteric | Decoction | Oral |  |
|  |  |  |  |  | disorder |  |  |  |
|  |  |  |  |  | Boil | Paste | Topical | 19.5 |
|  |  |  |  |  | Cancer | Decoction | Oral |  |
|  |  |  |  |  | Neuralgia | Decoction | Oral | 1.2 |
|  |  |  |  |  | Skin disease | Decoction | Oral |  |
|  |  |  |  |  |  | Maceration, paste | Topical |  |
|  |  |  |  | Root bark | Boil | Maceration, paste | Topical |  |
|  |  |  |  |  | Burn | Maceration, paste | Topical | 1.2 |
|  |  |  |  |  | Gastric cancer | Decoction | Oral | 3.7 |
|  |  |  |  |  | Pus | Maceration, paste | Topical |  |
|  |  |  |  | Stem | Furuncle | Maceration, paste | Topical |  |
| *Undaria pinnatifida* (Harvey) | KH5182 | Alariaceae | Miyeok | Thallus | Lumbago | Raw | Oral | 14.3 |
| Suringar |  |  |  |  | Puerperalism | Boiling | Oral | 85.7 |
| *Vigna radiata* (L.) Wilczek | KH5183 | Fabaceae | Nokdu | Seed | Pesticide | Grinding | Oral | 100.0 |
|  |  |  |  |  | detoxification |  |  |  |
| *Viola mandshurica* W. | KH5184 | Violaceae | Jebikkot | Aerial part | Hemostasis | Maceration, paste | Topical | 100.0 |
| Becker |  |  |  | Whole plant | Hemostasis | Maceration, paste | Topical |  |
| *Viscum coloratum* (Kom.) | KH5185 | Santalaceae | Gyeousali | Whole plant | Musculoskeletal | Decoction | Oral | 100.0 |
| Ohwi |  |  |  |  | disorders |  |  |  |
| *Vitex rotundifolia* L.f. | KH5186 | Lamiaceae | Sunbiginamu | Fruit | Facial nerve | Stuffing of a pillow | Topical | 3.3 |
|  |  |  |  |  | paralysis |  |  |  |
|  |  |  |  |  | Headache | Stuffing of a pillow | Topical | 66.7 |
|  |  |  |  |  | Paralysis | Decoction | Oral | 16.7 |
|  |  |  |  |  |  | Stuffing of a pillow | Topical |  |
|  |  |  |  |  | Vomiting | Decoction | Oral | 13.3 |
| *Xanthium strumarium* L. | KH5187 | Asteraceae | Dokkomari | Leaf | Cradle cap | Burning, powder | Topical | 80.0 |
|  |  |  |  | Fruit | Facial nerve | Decoction | Oral | 20.0 |
|  |  |  |  |  | paralysis |  |  |  |
| *Zanthoxylum piperitum* (L.) | KH5188 | Rutaceae | Chopinamu | Fruit | Indigestion | Decoction | Oral | 4.2 |
| DC. |  |  |  |  | Leg pain | Decoction | Oral | 33.3 |
|  |  |  |  |  | Lumbago | Decoction | Oral | 12.5 |
|  |  |  |  |  | Neuralgia | Decoction | Oral | 16.7 |
|  |  |  |  | Leaf | Detoxification | Drying, rubbing | Oral | 33.3 |
|  |  |  |  |  |  | Raw | Oral |  |
|  |  |  |  | Root | Leg pain | Decoction | Oral |  |
|  |  |  |  |  | Lumbago | Decoction | Oral |  |
|  |  |  |  | Stem | Leg pain | Decoction | Oral |  |
|  |  |  |  |  | Neuralgia | Decoction | Oral |  |
| *Zanthoxylum planispinum* | KH5189 | Rutaceae | Gaesancho | Root | Neuralgia | Taffy | Oral | 100.0 |
| Siebold & Zucc. |  |  |  |  |  |  |  |  |
| *Zea mays* L. | KH5190 | Poaceae | Oksusu | Style | Diuresis | Tea | Oral | 10.0 |
|  |  |  |  |  | Pollakiuria | Tea | Oral | 40.0 |
|  |  |  |  |  | Prostate disease | Decoction | Oral | 50.0 |
| *Zingiber mioga* Roscoe | KH5191 | Zingiberaceae | Yangha | Leaf sheath | Allergic contact | Maceration | Topical | 100.0 |
|  |  |  |  |  | dermatitis |  |  |  |
|  |  |  |  | Leaf | Allergic contact | Maceration | Topical |  |
|  |  |  |  |  | dermatitis |  |  |  |
| *Zingiber officinale* Roscoe | KH5192 | Zingiberaceae | Saenggang | Leaf | Bruise | Maceration, paste | Topical | 11.8 |
|  |  |  |  | Rhizome | Cold and flu | Decoction | Oral | 5.9 |
|  |  |  |  |  | Common cold | Decoction | Oral | 58.8 |
|  |  |  |  |  |  | Simmer | Oral |  |
|  |  |  |  |  | Cough | Decoction | Oral | 8.8 |
|  |  |  |  |  | Gastroenteric | Simmer | Oral | 8.8 |
|  |  |  |  |  | disorder |  |  |  |
|  |  |  |  |  | Neuralgia | Decoction | Oral | 5.9 |
| *Zizyphus jujuba* var. *inermis* | KH5193 | Rhamnaceae | Daechunamu | Fruit | Cold and flu | Decoction | Oral | 6.1 |
| (Bunge) Rehder |  |  |  |  | Common cold | Decoction | Oral | 75.8 |
|  |  |  |  |  |  | Simmer | Oral |  |
|  |  |  |  |  |  | Tea | Oral |  |
|  |  |  |  |  | Cough | Decoction | Oral | 9.1 |
|  |  |  |  |  | Gastroenteric | Simmer | Oral | 9.1 |
|  |  |  |  |  | disorder |  |  |  |
